# Supplementary material for: The plant unique ESCRT component FREE1 regulates autophagosome closure
Source: Nat Commun. 2023 Mar 30;14:1768. doi: 10.1038/s41467-023-37185-6 (PMC10063618; doi:10.1038/s41467-023-37185-6)
Supplement: Supplementary file 1 — Supplementary Information [file 41467_2023_37185_MOESM1_ESM.pdf]

Supplementary Materials for

The Plant Unique ESCRT Component FREE1 Regulates Autophagosome Closure

Yonglun Zeng#, Baiying Li#, Shuxian Huang#, Hongbo Li, Wenhan Cao, Yixuan Chen, Guoyong Liu, Zhenping Li, Chao Yang, Lei Feng, Jiayang Gao, Sze Wan Lo, Jierui Zhao, Jinbo Shen, Yan Guo, Caiji Gao, Yasin Dagdas, and Liwen Jiang*

* Corresponding author. Email: ljiang@cuhk.edu.hk (L.J.)

# These authors contributed equally to this work.

This PDF file includes:

Supplementary Figures 1 to 31

Other Supplementary Material for this manuscript includes the following:

Supplementary Movies 1 to 6 (.mov)

Supplementary Data 1 and 2 (.xlsx)

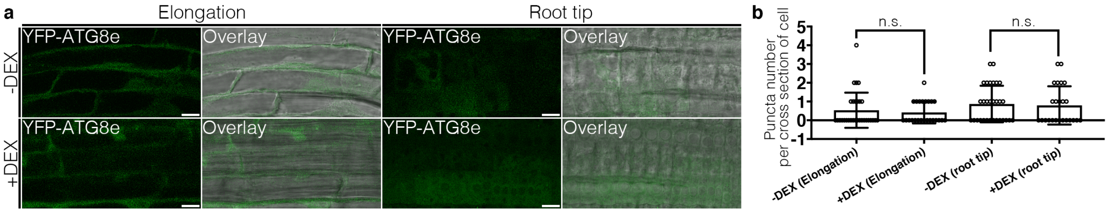

Supplementary Fig 1. DEX treatment does not affect autophagosome formation in plants. a Confocal imaging of YFP-ATG8e upon mock or DEX treatments. Transgenic seedlings expressing YFP-ATG8e were subjected to mock or DEX treatments for at least 8hrs before confocal laser scanning microscopy (CLSM) observation. Scale bar, 10 μm. **b** Quantification analysis of the puncta number per cross-cell section with or without DEX treatments in the root tip or elongation zone shown in **a**. Means ± SD; n=30 (-DEX, elongation), n=24 (+DEX, elongation), n=30 (-DEX, root tip), n=24 (+DEX, root tip) individual cross-cell section per experimental group, two-tailed unpaired t test; n.s., not significant. All the imaging analysis was repeated at least for three times with similar results.

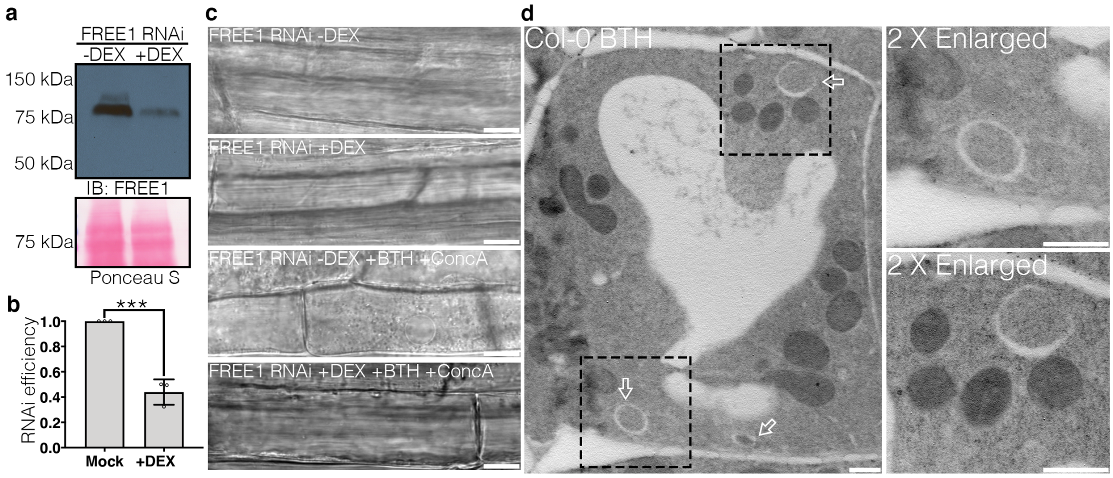

Supplementary Fig 2. FREE1 knockdown affects autophagosome closure and vacuolar delivery of autophagosomes. **a** The dexamethasone (DEX)-inducible FREE1 RNAi (*DEX::RNAi-FREE1*) mutants were subjected to mock or DEX treatments for 8hrs, followed by protein extraction and immunoblotting by anti-FREE1. **b** Quantification analysis of the knock-down efficiency of FREE1 shown in **a**. Means ± SD; n=3 individual experiments, two-tailed unpaired t test, ***p<0.001. **c** Bright-field imaging visualization of vacuolar accumulation of autophagic bodies in FREE1 RNAi (*DEX::RNAi-FREE1*) mutants upon mock or BTH + ConcA treatments with or without DEX inductions for 8hrs. Scale bar, 10 µm. **d** TEM analysis of the closed autophagosomes in high pressure freezing/frozen Col-0 *Arabidopsis* root tips upon BTH treatment for 8hrs. Arrows indicated examples of closed autophagosomes. Black dash boxes indicate the 2 X Enlarged areas. Scale bars, 500 nm. All the imaging analysis and immunoblots were repeated at least for three times with similar results.

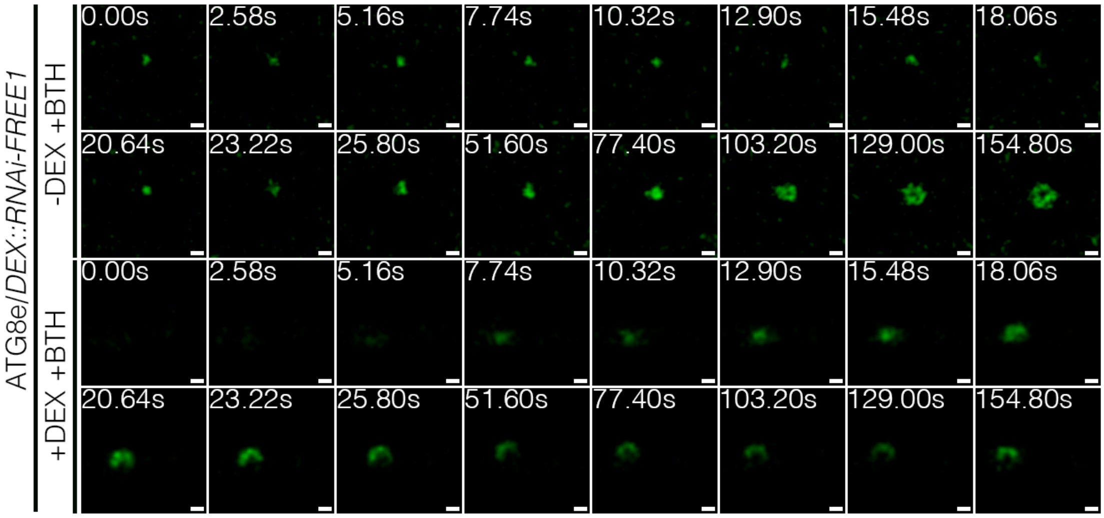

Supplementary Fig 3. Formation and dynamic of autophagosomal structures in *FREE1* RNAi mutants upon autophagic induction. Time-lapse imaging of transgenic *Arabidopsis* seedlings expressing YFP-ATG8e in *DEX::RNAi-FREE1* mutants upon BTH-induced autophagy with or without DEX treatments for 8hrs. Scale bars, 1 μm. All the imaging analysis was repeated at least for three times with similar results.

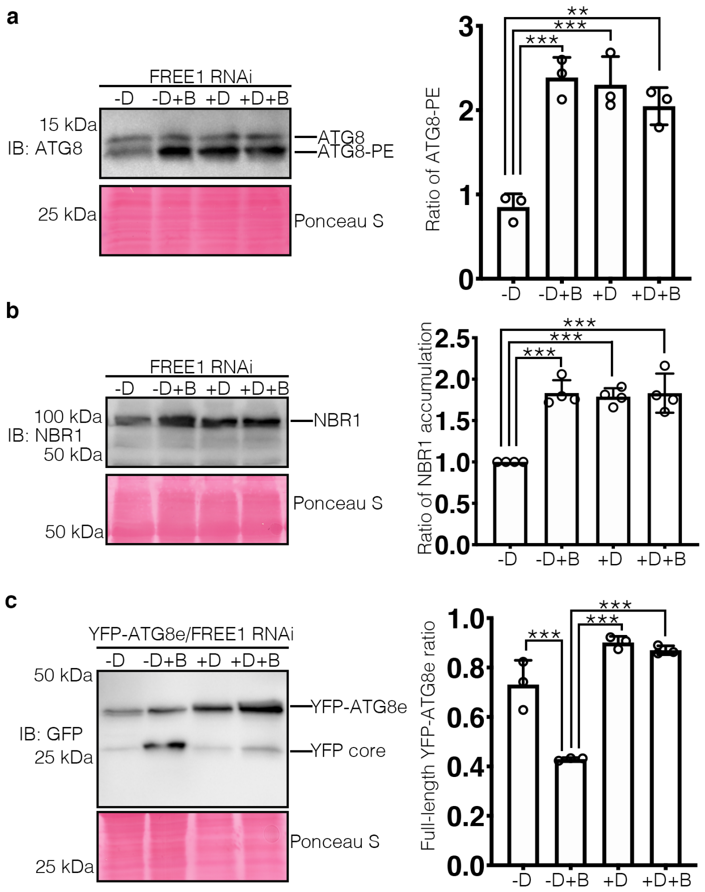

Supplementary Fig 4. Biochemical analysis of autophagosome progression in FREE1 knock-down mutants. a The dexamethasone (DEX)-inducible FREE1 RNAi (*DEX::RNAi-FREE1*) mutants were treated without DEX (-D), without DEX with BTH (-D+B), with DEX (+D), or with DEX and BTH (+D+B) for 8hrs, followed by protein extraction and subsequent detection of the ATG8 lipidation using Atg8 antibody. Quantification analysis of the ATG8-PE ratio in individual experimental group. Means ± SD; n=3 individual experiments, one-way analysis of variance (ANOVA), followed by Tukey's multiple test; **p<0.01; ***p<0.001. **b** The dexamethasone (DEX)-inducible FREE1 RNAi (*DEX::RNAi-FREE1*) mutants were treated without DEX (-D), without DEX with BTH (-D+B), with DEX (+D), or with DEX and BTH (+D+B) for 8hrs, followed by protein extraction and immunoblotting detection of the endogenous NBR1 accumulation by NBR1 antibody. Quantification analysis of the NBR1 ratio in each group comparing with the control (-D) in individual experimental group. Means ± SD; n=4 individual experiments, one-way analysis of variance (ANOVA), followed by Tukey's multiple test; ***p<0.001. **c** Transgenic *Arabidopsis* seedlings expressing YFP-ATG8e in FREE1 RNAi (*DEX::RNAi-FREE1*) mutants were subjected to mock (-D), without DEX with BTH

(-D+B), DEX (+D), or DEX and BTH (+D+B) treatments for 8hrs, followed by protein extraction and immunoblotting detection by GFP antibody. Quantification analysis of the full-length YFP-ATG8e ratio in individual experimental group. Means ± SD; n=3 individual experiments, one-way analysis of variance (ANOVA), followed by Tukey's multiple test; ***$p<0.001$. All the immunoblots were repeated at least for three times with similar results.

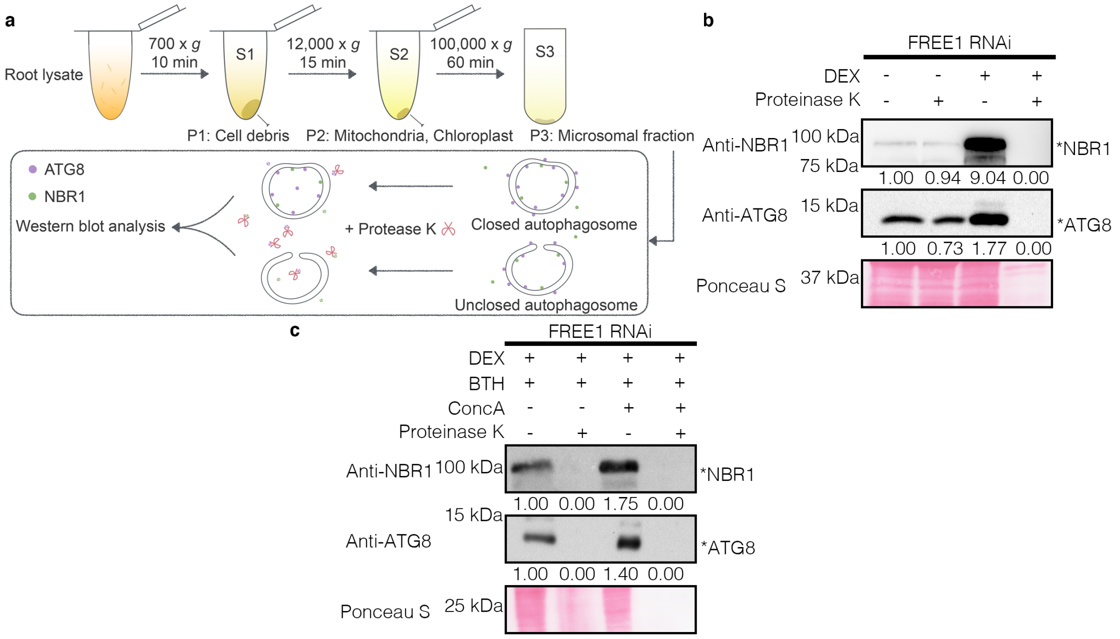

Supplementary Fig 5. Protease protection assay of the enriched autophagosomal structures in FREE1 depletion mutants. a Diagram showing the procedure for autophagosomal membranes enrichment used for the protease protection assay in plants. Roots from *Arabidopsis* seedlings were resected and proceeded for low-speed (10 min, 700 x *g*), high-speed (15 min, 12,000 x *g*), and ultra-speed (60 min, 100,000 x *g*) centrifugation to enrich the microsomal fraction containing autophagosomal membranes for western blot analysis using antibodies against autophagy-related proteins. **b** The enriched autophagosomal membranes isolated from the dexamethasone (DEX)-inducible FREE1 RNAi (*DEX::RNAi-FREE1*) mutants with or without DEX treatments were proceeded for proteinase K digestions, followed by protein extraction and subsequent western blot analysis using NBR1 and Atg8 antibodies. **c** The enriched autophagosomal membranes isolated from the dexamethasone (DEX)-inducible FREE1 RNAi (*DEX::RNAi-FREE1*) mutants upon DEX and BTH inductions with or without ConcA treatments were proceeded for proteinase K digestions, followed by protein extraction and subsequent western blot analysis using NBR1 and Atg8 antibodies. All the immunoblots were repeated at least for three times with similar results.

a

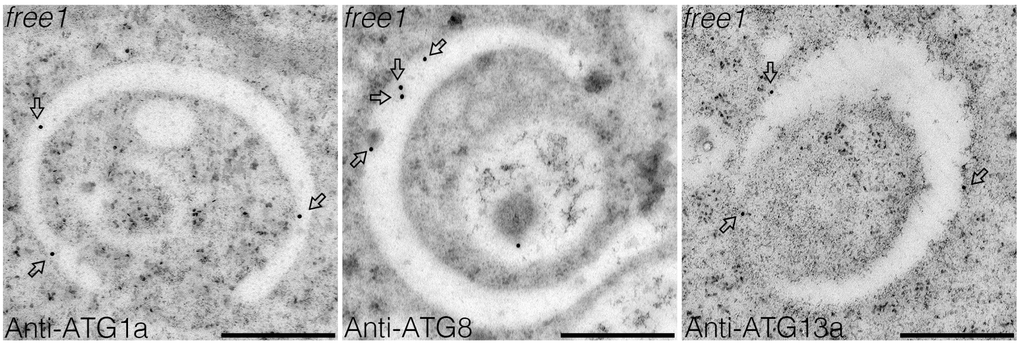

b

Quantitative analysis of TEM immunolocalization signal density[a] in *free1*		
Sample[b]	unclosed autophagosome-like structures No. Gold/μm² ±SD	non-unclosed autophagosome-like structures No. Gold/μm² ±SD
Anti-ATG1a	4.6 ± 1.4	0.9 ± 1.0
Anti-ATG8	4.5 ± 1.8	0.3 ± 0.7
Anti-ATG13a	4.3 ± 1.2	0.3 ± 0.5

[a] Signal density is expressed as the number of gold particles/μm² ±SD
[b] At least 10 cells were observed for quantification

Supplementary Fig 6. Immunogold-TEM analysis of ATG machinery on the unclosed autophagosomal structures in *free1* mutants. a Immunogold-TEM analysis of high-pressure freezing/frozen substituted homozygous *free1* T-DNA insertional mutant root tips upon BTH treatment for 8hrs using antibodies against autophagy-related proteins including ATG1a, ATG8, and ATG13a. Arrows indicate examples of the gold-particle labeling on the unsealed autophagosomal structures in *free1* mutants. Scale bars, 500 nm. **b** Quantification analysis of the gold-particle number on the autophagosomal structures shown in **a**.

a

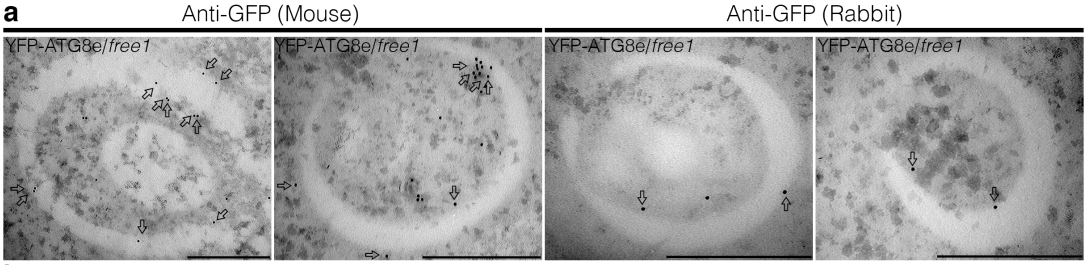

b

Quantitative analysis of TEM immunolocalization signal density[a] in YFP-ATG8e/*free1*

Sample[b]	unclosed autophagosome-like structures No. Gold/μm^2 ±SD	non-unclosed autophagosome-like structures No. Gold/μm^2 ±SD
Anti-GFP (Mouse)	7.0 ± 4.3	1.9 ± 1.7
Anti-GFP (Rabbit)	4.2 ± 2.2	0.9 ± 0.7

[a] Signal density is expressed as the number of gold particles/μm^2 ±SD
[b] At least 10 cells were observed for quantification

Supplementary Fig 7. Immunogold-TEM analysis of the unclosed autophagosomal structures in YFP-ATG8e/*free1* lines using GFP antibodies. a Immunogold-TEM analysis of high-pressure freezing/frozen substituted homozygous *free1* T-DNA insertional mutant root tips expressing YFP-ATG8e upon BTH treatment for 8hrs using GFP antibodies (anti-mouse or anti-rabbit). Arrows indicate examples of the gold-particle labeling on the unsealed autophagosomal structures in *free1* mutants. Scale bars, 500 nm. **b** Quantification analysis of the gold-particle number on the autophagosomal structures shown in **a**.

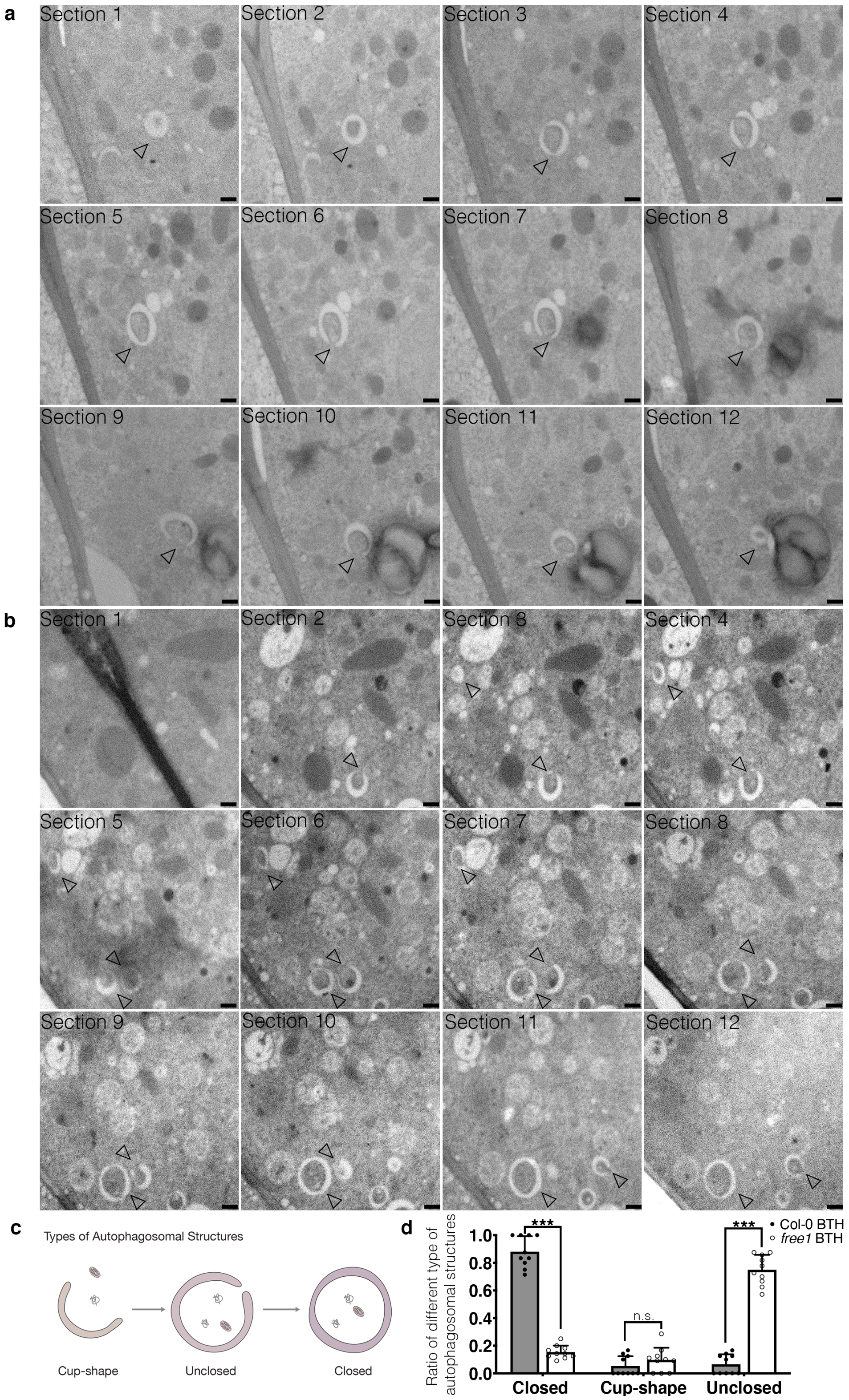

Supplementary Fig 8. Serial-2D TEM analysis of autophagosomes in *free1* mutants. a b Serial-2D TEM analysis of high-pressure freezing/frozen substituted root cells of *free1* mutant upon BTH treatment for 8hrs. Continuous thin-sections were collected for 2D-TEM analysis. Arrowheads indicate examples of the unsealed autophagosomal structures under conventional TEM. Scale bars, 500 nm. **c** Diagram

showing the types of autophagosomal structures during autophagosome formation. **d** Quantification analysis of the ratio of the closed, cup-shape, and unclosed autophagosomal structures in Col-0 or *free1* mutants upon BTH treatment using serial-2D TEM in **a** and **b**. Means ± SD; n=10 (Col-0) and n=10 (*free1*) individual cells per experimental group, two-way analysis of variance (ANOVA), followed by Šídák's multiple comparisons test; ***p<0.001; n.s., not significant.

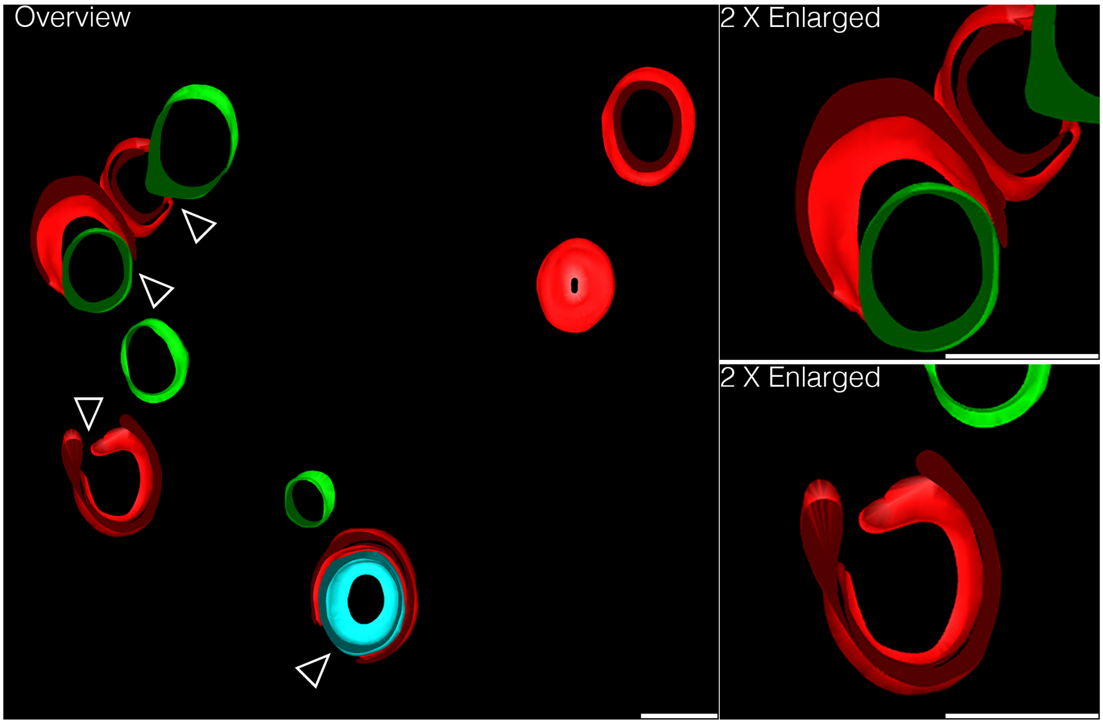

Supplementary Fig 9. 3D-tomography overview of unclosed autophagosomes in *free1* mutant. Model of 3D-tomography showing the unclosed autophagosomal structures in homozygous *free1* T-DNA insertional mutant upon BTH treatment. Left panel, model showing the overview of the 3D-tomography. Right panel, 2 X Enlarged of the overview. Arrowheads indicate examples of the unsealed autophagosomal structures. Scale bars, 500 nm. The red color indicates the autophagosomal structures, while the blue and green color indicate the membrane-bound organelles.

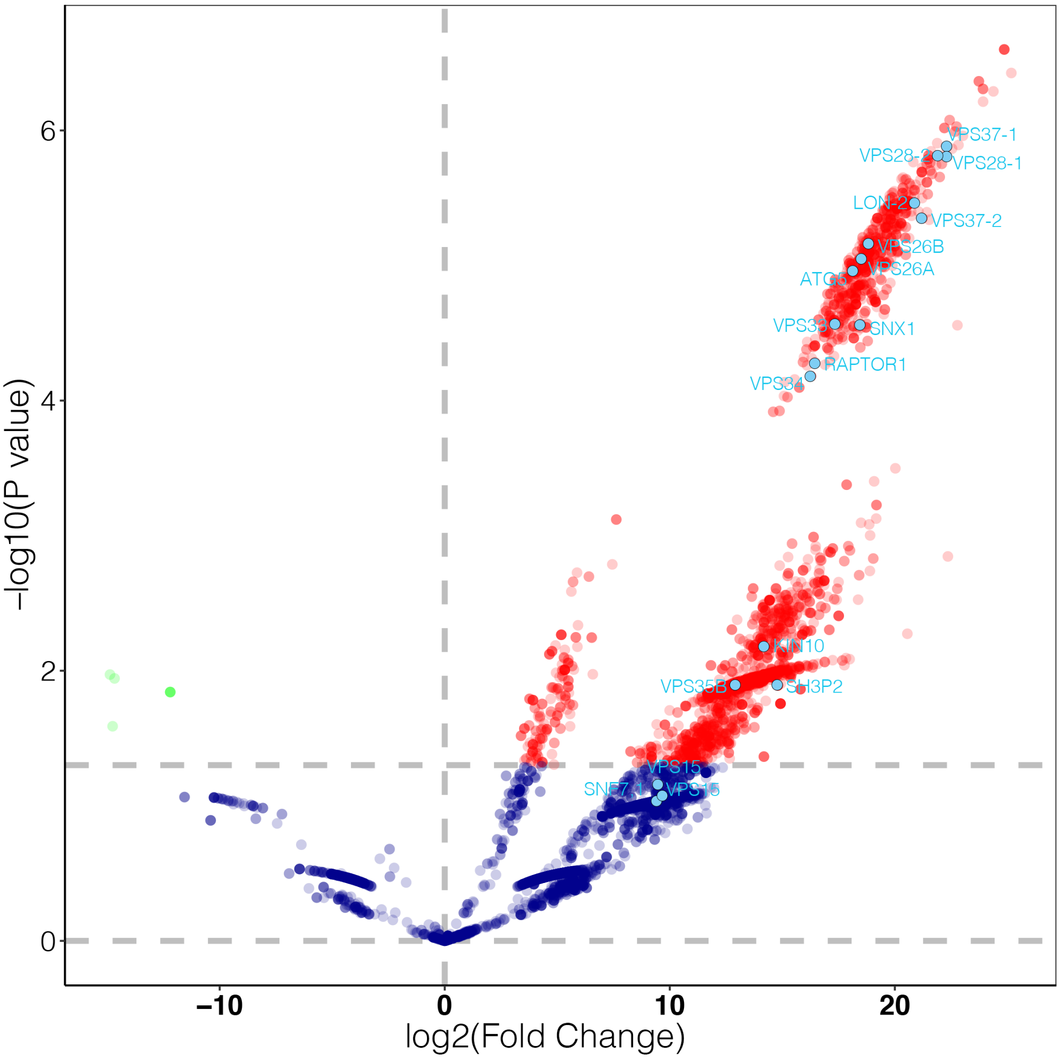

Supplementary Fig 10. Identification of the GFP-FREE1 interactors by affinity purification-mass spectrometry (AP-MS) analysis. The IBAQ values of the enriched proteins from four individual GFP-FREE1 or GFP pull-down groups were calculated by the MaxQuant and proceeded for the volcano plot analysis to compare the proteins IBAQ values between GFP-FREE1 groups and GFP groups for FREE1 interactors identification. Red dots represent the significantly enriched proteins in GFP-FREE1 group (P value≤0.05), green dots represent the significantly enriched proteins in GFP group (P value≤0.05), and blue dots represent the non-significantly enriched proteins in GFP-FREE1 group (P value>0.05).

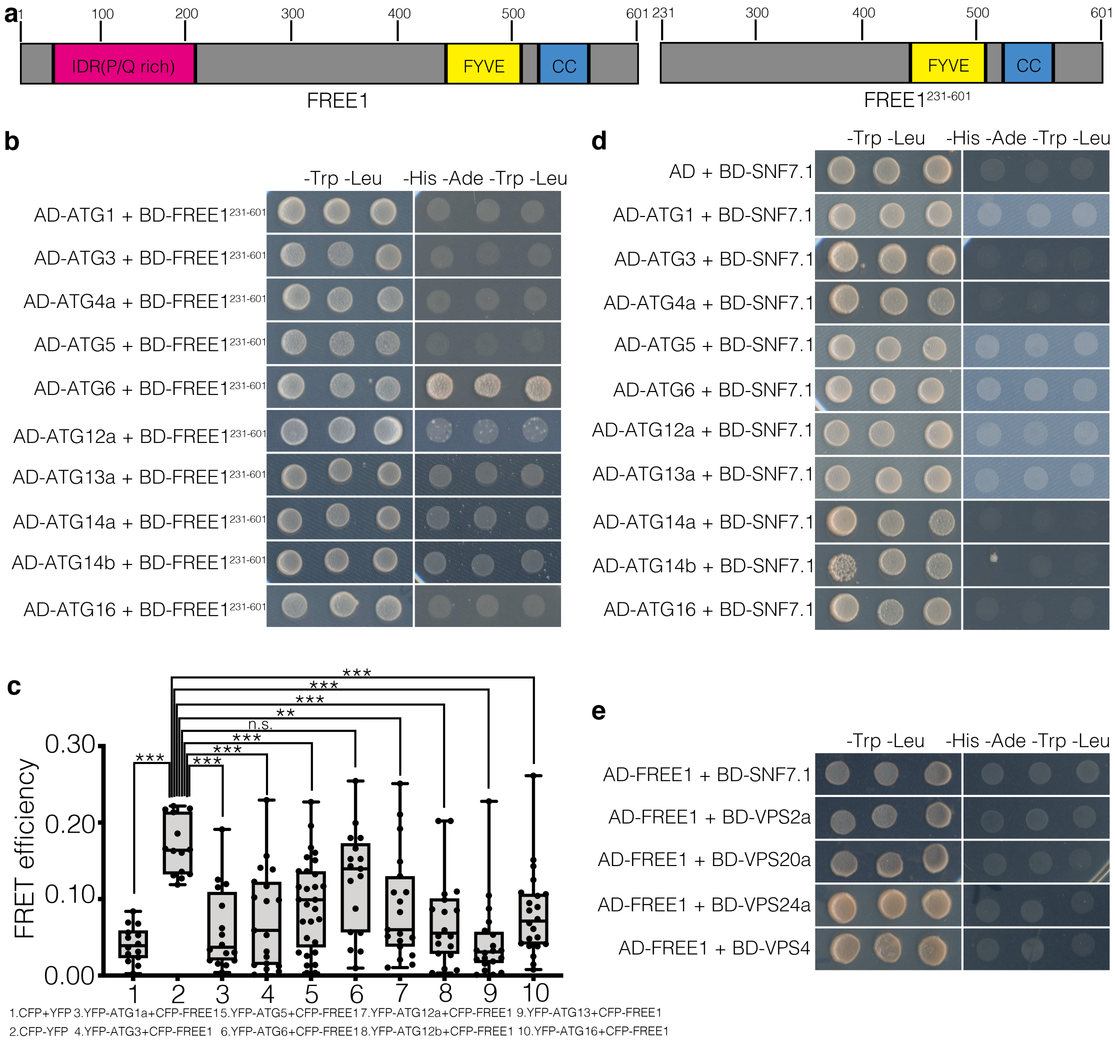

Supplementary Fig 11. FREE1 does not show direct interaction with other ATG machinery and ESCRT-III components. a Schematic diagram of FREE1 and FREE1$^{231\text{-}601}$ (the truncated FREE1 without self-activation on BD vector) used for Y2H. **b** Y2H analysis of BD-FREE1$^{231\text{-}601}$ with ATG proteins as indicated. **c** FRET analysis between CFP-FREE1 and various YFP fusions of the ATG machinery in *Arabidopsis* protoplasts. *Arabidopsis* protoplasts were transfected with CFP and YFP (negative control), CFP-YFP (positive control), CFP-FREE1 and YFP-ATG1a, CFP-FREE1 and YFP-ATG3, CFP-FREE1 and YFP-ATG5, CFP-FREE1 and YFP-ATG6, CFP-FREE1 and YFP-ATG12a, CFP-FREE1 and YFP-ATG12b, CFP-FREE1 and YFP-ATG13, or CFP-FREE1 and YFP-ATG16, followed by cultured for 24hrs before FRET analysis. Means ± SD; n=14 (CFP/YFP), n=14 (CFP-YFP), n=16 (CFP-FREE1/YFP-ATG1a), n=19 (CFP-FREE1/YFP-ATG3), n=29 (CFP-FREE1/YFP-ATG5), n=17 (CFP-FREE1/YFP-ATG6), n=19 (CFP-FREE1/YFP-ATG12a), n=18 (CFP-FREE1/YFP-ATG12b), n=20 (CFP-FREE1/YFP-ATG13), and n=22 (CFP-FREE1/YFP-ATG16) individual puncta per experimental group, one-way analysis of

variance (ANOVA), followed by Dunnett's multiple comparisons test; **$p<0.01$; ***$p<0.001$; n.s., not significant. The middle lines of the boxes represent the medians of datasets. The upper and bottom lines of the boxes are respectively the upper quantile and the lower quantile of the data. The whiskers mark the upper and lower limits of these datasets, respectively. **d** Y2H analysis of BD-SNF7.1 with ATG proteins. **e** Y2H analysis of AD-FREE1 with ESCRT-III machinery.

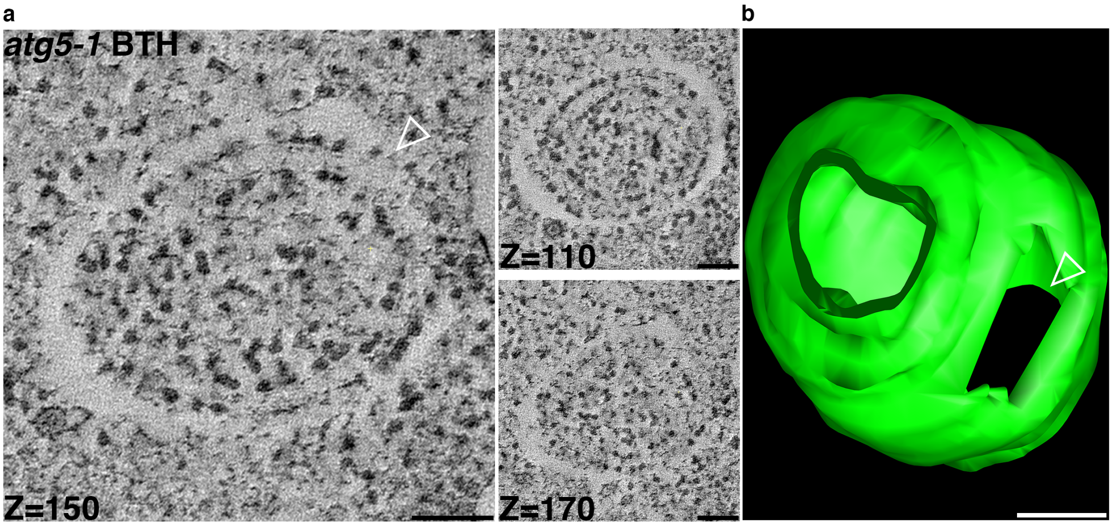

Supplementary Fig 12. 3D-TEM observation of the unclosed autophagosomes in *atg5-1* mutants. a 3D electron tomography analysis of unclosed autophagosomal structures in *atg5-1* mutants upon BTH treatment for 8hrs. Arrowhead indicates the unsealed membrane on the autophagosome. Scale bars, 100 nm. **b** 3D model of the tomography. Arrowhead indicates the "open" site on the unsealed autophagosomal structure. Scale bars, 100 nm. The green color indicates the autophagosomal structure. All the imaging analysis was repeated at least for three times with similar results.

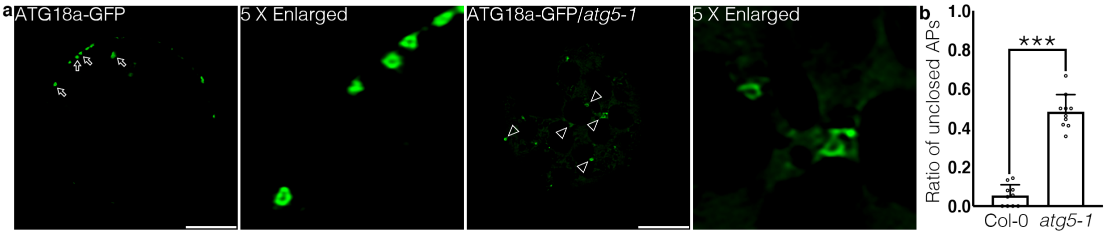

Supplementary Fig 13. Accumulation of the unclosed ATG18a-positive structures in *atg5-1* mutants. a Transient expression and confocal imaging analysis of the ATG18a-GFP positive structures in *Arabidopsis* leaf protoplasts isolated from wild-type plants or *atg5-1* mutants. The transfected leaf protoplasts were cultured for 24hrs before CLSM observation and image deconvolution. Scale bars, 10 µm. Arrows and arrowheads indicated examples of closed and unsealed autophagosomal structures, respectively. **b** Quantification analysis of the unclosed autophagosomes (APs) ratio in protoplasts isolated from wild-type plants or *atg5-1* mutant background shown in **a**. Means ± SD; n=10 protoplast cells for each group, two-tailed unpaired t test; ***p<0.001. All the imaging analysis was repeated at least for three times with similar results.

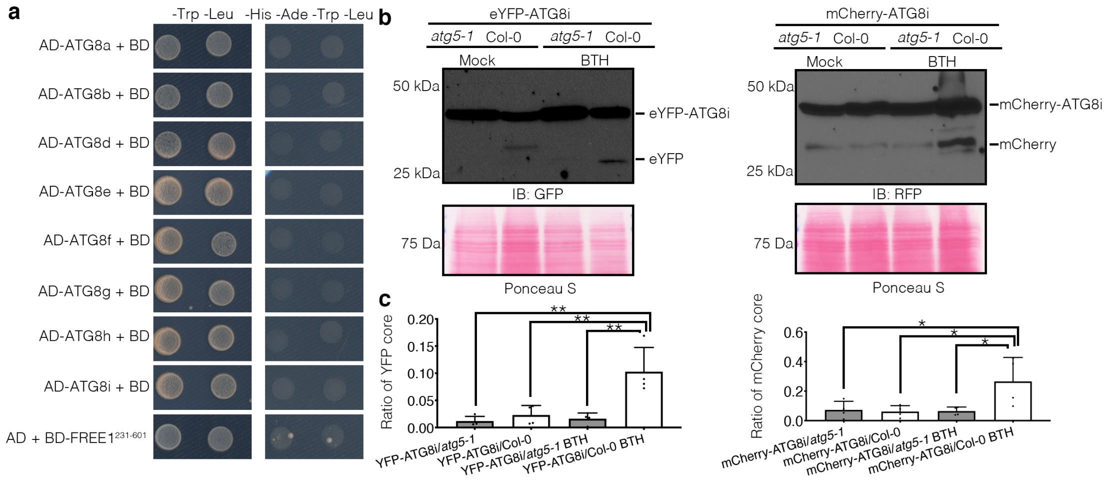

Supplementary Fig 14. ATG5 is essential for ATG8i-positive autophagic flux. a Y2H analysis of empty BD vector with ATG8 isoforms. **b** 5-day-old transgenic *Arabidopsis* seedlings expressing eYFP-ATG8i or mCherry-ATG8i in *atg5-1* mutant background were treated with or without BTH for 8hrs, followed by protein extraction and subsequent immunoblot analysis using GFP or RFP antibodies to detect ATG8 vacuolar turnover. **c** Quantification analysis of the YFP or RFP core ratio in individual experimental group shown in **b**. Means ± SD; n=4 individual experiments, one-way analysis of variance (ANOVA), followed by Tukey's multiple test; *p<0.05; **p<0.01. All the immunoblots were repeated at least for three times with similar results.

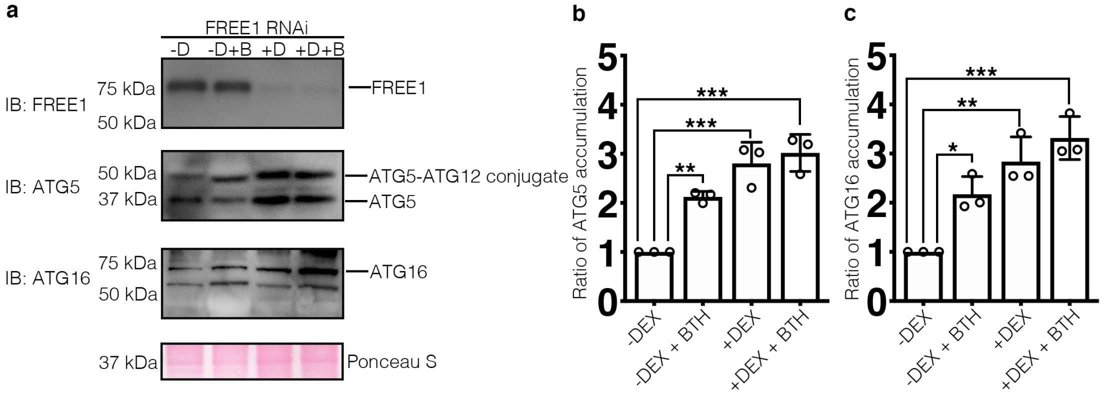

Supplementary Fig 15. Accumulation of ATG conjugation system components in FREE1 depletion mutants. **a** Dexamethasone (DEX)-inducible FREE1 RNAi (*DEX::RNAi-FREE1*) mutants were treated without DEX (-D), without DEX with BTH (-D+B), with DEX (+D), or with DEX and BTH (+D+B), followed by protein extraction and subsequent western blot analysis using antibodies against ATG conjugation system components including ATG5 and ATG16. **b** Quantification of ATG5-ATG12 conjugate accumulation in **a**. Means ± SD; n=3 individual experiments, one-way analysis of variance (ANOVA), followed by Tukey's multiple test; **p<0.01; ***p<0.001. **c** Quantification of ATG16 accumulation in **a**. Means ± SD; n=3 individual experiments, one-way analysis of variance (ANOVA), followed by Tukey's multiple test; *p<0.05; **p<0.01; ***p<0.001. All the immunoblots were repeated at least for three times with similar results.

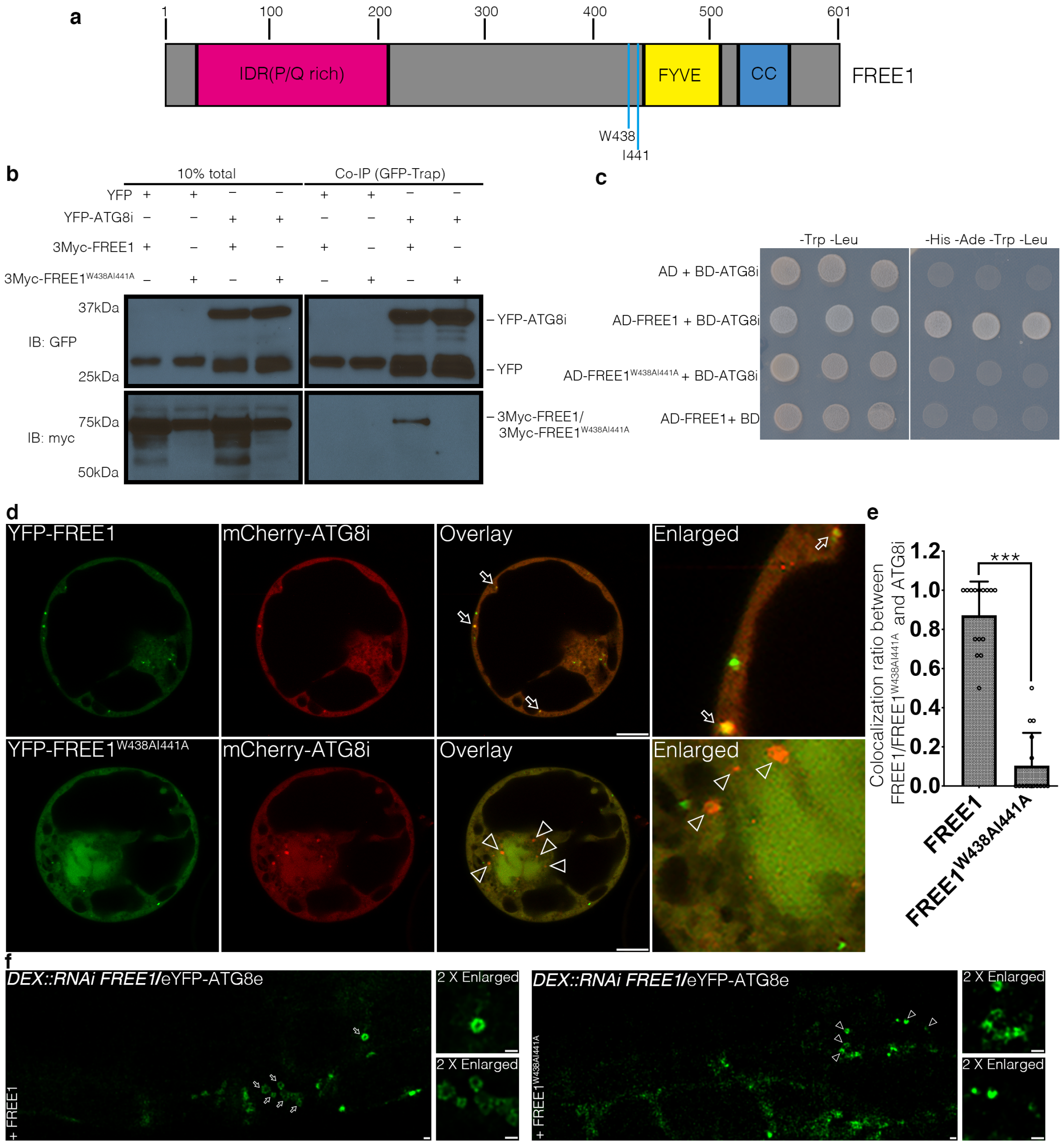

Supplementary Fig 16. FREE1 interacts with ATG8 via a canonical AIM motif. a Diagram showing the predicted AIM motif on FREE1 protein. **b** GFP-Trap and co-IP analysis of 3Myc-FREE1 and its AIM motif mutant 3Myc-FREE1^{W438AI441A} with YFP-ATG8i using *Arabidopsis* protoplasts. *Arabidopsis* protoplasts co-expressing 3Myc-FREE1 or 3Myc-FREE1^{W438AI441A} with YFP-ATG8i or YFP only were subjected to protein extraction and IP with GFP-Trap, followed by immunoblotting with indicated antibodies. **c** Y2H analysis of AD-FREE1 or AD-FREE1^{W438AI441A} with BD-ATG8i isoform. **d** Transient co-expression of YFP-FREE1 or YFP-FREE1^{W438AI441A} with mCherry-ATG8i in *Arabidopsis* protoplasts, the transfected protoplasts were cultured for 24hrs before CLSM observation. Arrows and arrowheads indicated the colocalized and separated puncta, respectively. Scale bars, 10 μm. **e** Quantification analysis of the colocalization ratio between YFP-FREE1 or

YFP-FREE1^{W438AI441A} and mCherry-ATG8i shown in **d**. Means ± SD; n=15 cells for each group, two-tailed unpaired t test; ***p<0.001. **f** Overexpression of the FREE1^{W438AI441A} in *DEX::RNAi-FREE1* mutants cannot rescue the autophagosome closure. Transgenic *Arabidopsis* seedlings expressing YFP-ATG8e in *DEX::RNAi-FREE1* mutants were subjected to particle bombardment of FREE1 or FREE1^{W438AI441A}, followed by carbon starvation for at least 18hrs before CLSM observation and image deconvolution. Arrows and arrowheads indicated examples of closed and unsealed autophagosomal structures, respectively. Scale bar, 1 μm. All the imaging analysis and immunoblots were repeated at least for three times with similar results.

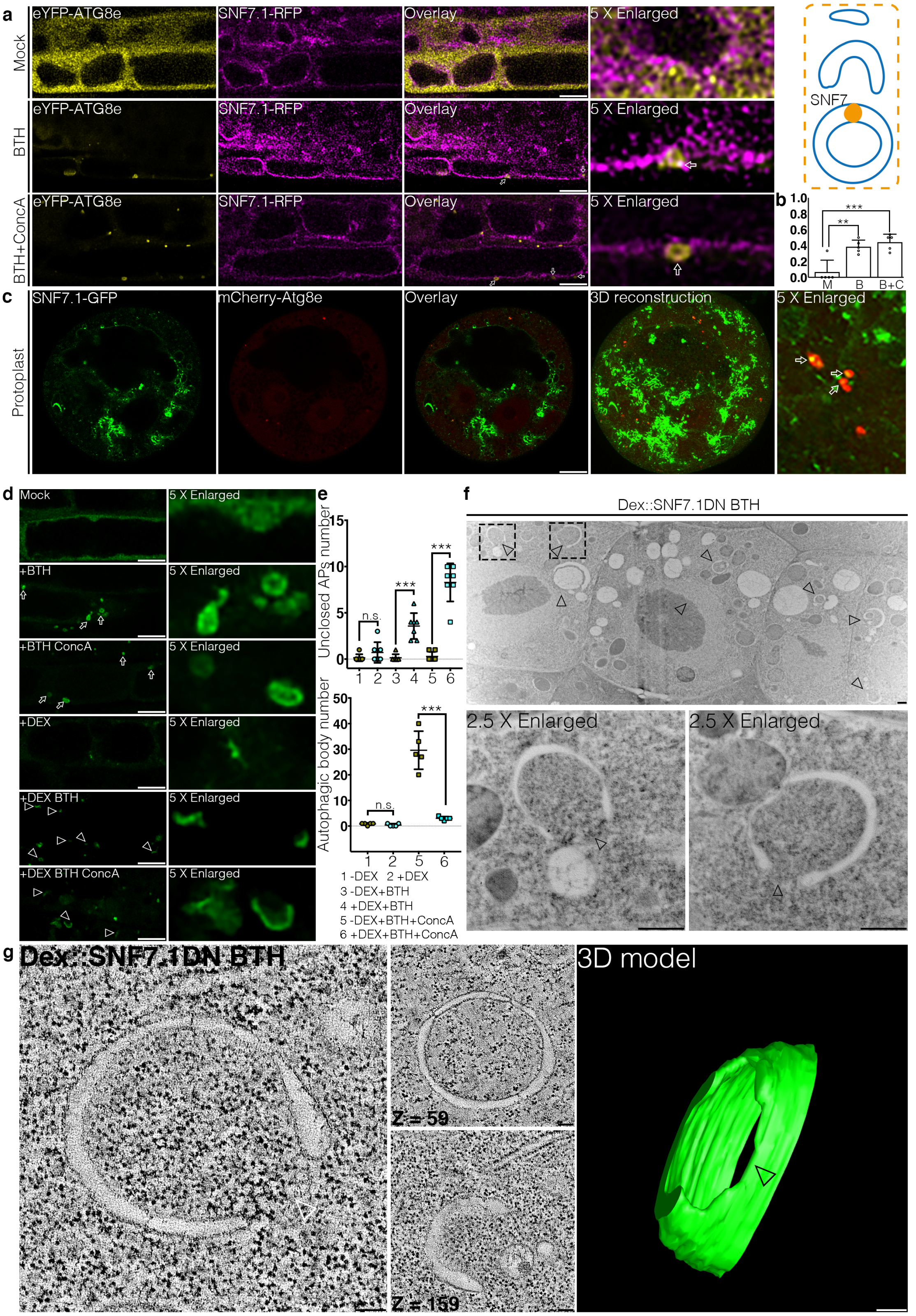

Supplementary Fig 17. The ESCRTIII complex component SNF7 is essential for autophagosome closure. a Transgenic *Arabidopsis* seedlings co-expressing eYFP-ATG8e and SNF7.1-RFP were subjected to mock or autophagy inductions using BTH, BTH and concanamycin A for 8hrs before CLSM observation and image deconvolution. Arrows indicated the tip-localized SNF7.1 on the autophagosomal

membrane. Scale bars, 10 μm. **b** Quantification analysis of the colocalization between SNF7.1 and ATG8e shown in **a**. Means ± SD; n=5 individual cells, one-way analysis of variance (ANOVA), followed by Tukey's multiple test; **p<0.01; ***p<0.001. M, Mock. B, BTH. C, ConcA. **c** *Arabidopsis* protoplasts were transfected with SNF7.1-GFP and mCherry-ATG8e for confocal observation. Arrows indicated examples of tip-localized SNF7.1 on the autophagosomal membrane. Scale bars, 10 μm. **d** 5-day-old transgenic *Arabidopsis* seedlings co-expressing *DEX::SNF7.1DN* with YFP-ATG8e were treated with mock, BTH, or BTH and ConcA, with or without DEX induction for 8hrs before confocal imaging and deconvolution. Arrows and arrowheads indicated examples of closed and unclosed autophagosomes, respectively. Scale bars, 10 μm. **e** Quantification analysis of unclosed autophagosomes (APs) (upper panel) and autophagic bodies inside the vacuoles (lower panel) shown in **c**. Means ± SD; n=7 (upper panel) and n=5 (lower panel) individual cross-cell section per experimental group, one-way analysis of variance (ANOVA), followed by Tukey's multiple test; ***p<0.001; n.s., not significant. **f** TEM analysis of high-pressure freezing/frozen substituted *DEX::SNF7.1DN* mutant root tips upon DEX and BTH treatment for 8hrs. Black dash boxes indicated the 2.5 X zoom in. Arrowheads indicated examples of unclosed autophagosomes. Scale bars, 500 nm. **g** 3D electron tomography analysis of autophagosomal structures in *DEX::SNF7.1DN* mutant upon BTH treatment for 8hrs. Left panel, individual slice of the 3D-tomography. Right panel, 3D model of the tomography. Arrowhead indicated the "open" site on the unsealed autophagosomal structures. Scale bars, 100 nm. The green color indicates the autophagosomal structure. All the imaging analysis was repeated at least for three times with similar results.

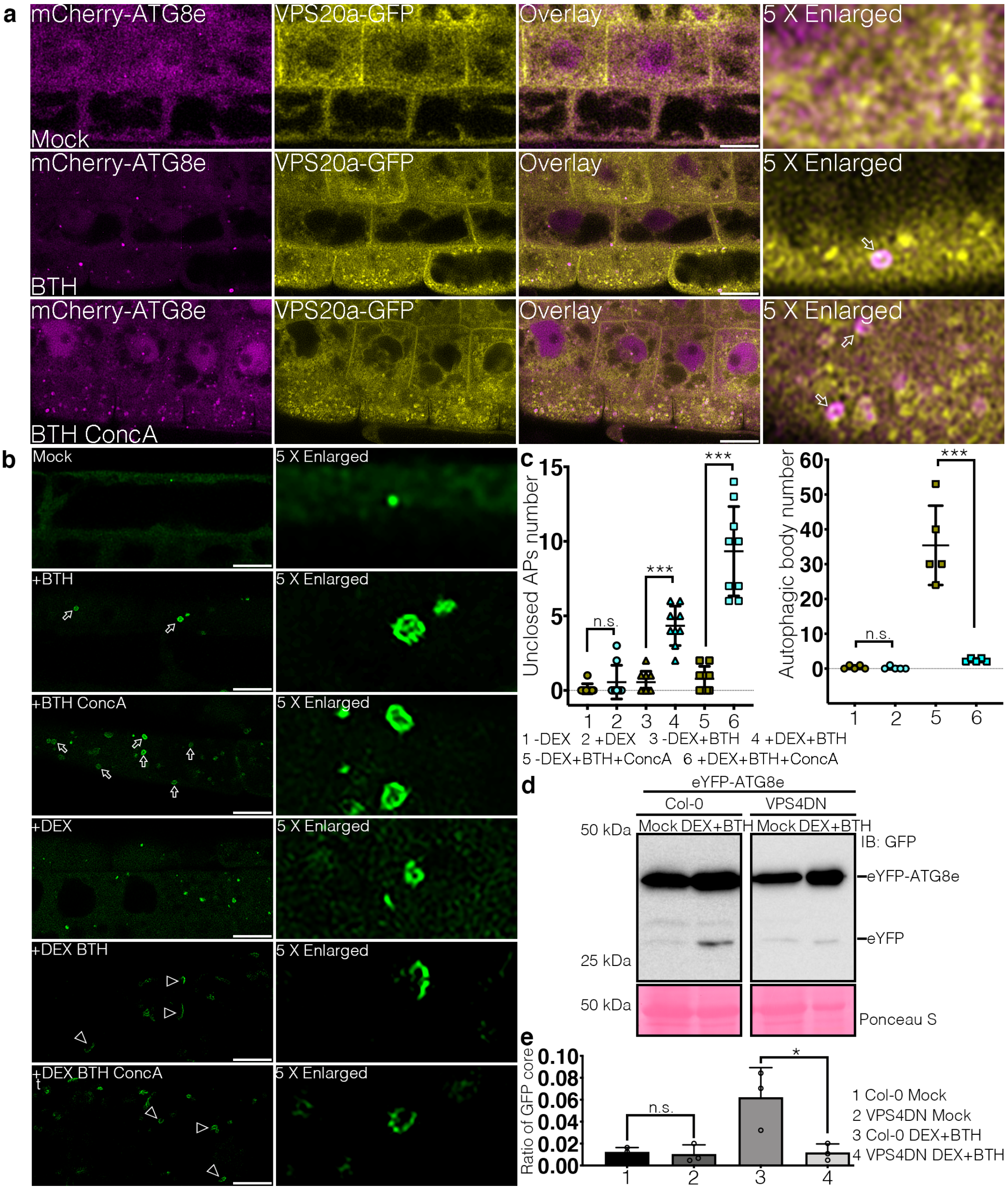

Supplementary Fig 18. Other components of ESCRTIII machinery also exhibit autophagosomal localization *in planta* upon autophagic inductions. a Transgenic *Arabidopsis* seedlings co-expressing mCherry-ATG8e and VPS20a-GFP were subjected to mock or autophagy inductions using BTH, BTH and ConcA for 8hrs before CLSM observation and images deconvolution. Arrows indicated examples of the tip-localized of VPS20a on the autophagosomal structures. Scale bars, 10 µm. **b** Transgenic *Arabidopsis* seedlings co-expressing *DEX::VPS4DN* with YFP-ATG8e were treated with mock, BTH, or BTH and ConcA, with or without DEX induction for 8hrs before CLSM imaging and deconvolution. Arrows indicated examples of closed autophagosomes, while arrowheads indicated examples of unclosed

autophagosomes. Scale bars, 10 μm. **c** Quantification analysis of unclosed autophagosomes (APs) (left panel) and autophagic bodies inside the vacuoles (right panel) shown in **b**. Means ± SD; n=9 (left panel) and n=5 (right panel) individual cross-cell section per experimental group, one-way analysis of variance (ANOVA), followed by Tukey's multiple test; ***p<0.001; n.s., not significant. **d** Transgenic *Arabidopsis* seedlings expressing eYFP-ATG8e in wild-type Col-0 or *DEX::VPS4DN* mutants were treated with or without BTH and DEX for 8hrs, followed by protein extraction and subsequent immunoblot analysis using GFP antibodies to detect ATG8 vacuolar turnover. **e** Quantification analysis of the GFP core ratio in individual experimental group shown in **d**. Means ± SD; n=3 individual experiments, one-way analysis of variance (ANOVA), followed by Tukey's multiple test; *p<0.05; n.s., not significant. All the imaging analysis and immunoblots were repeated at least for three times with similar results.

a

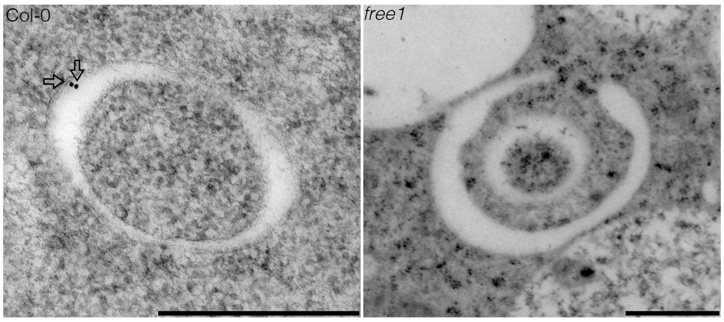

b

Quantitative analysis of TEM immunolocalization signal density[a] on autophagosome		
Sample[b]	Col-0 No. Gold±SD	*free1* No. Gold±SD
Anti-SNF7	2.1 ± 0.7	0.2 ± 0.4

[a] Signal density is expressed as the number of gold particles±SD per autophagosome
[b] At least 10 cells were observed for quantification

d

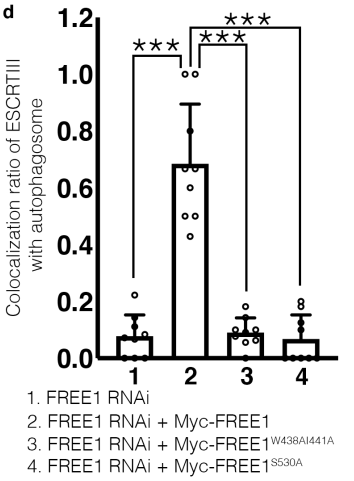

c

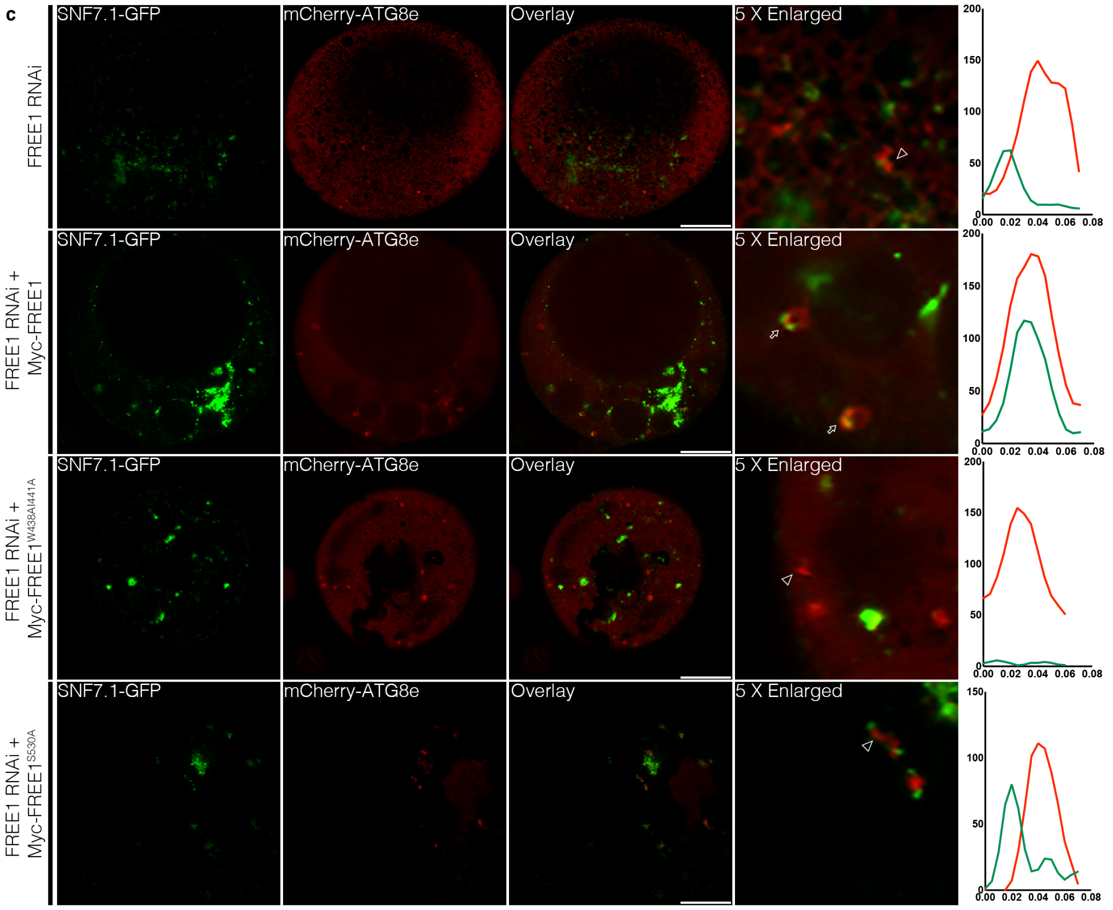

e

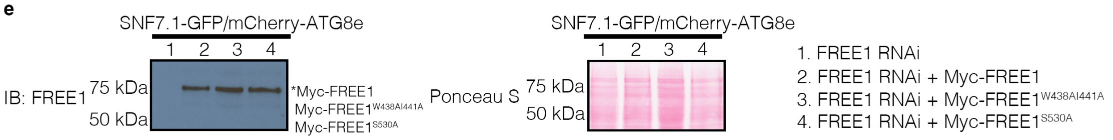

Supplementary Fig 19. Failure of ESCRTIII localization to phagophores in FREE1 depletion mutants. a Immunogold-TEM analysis of high-pressure freezing/frozen substituted wild-type Col-0 or homozygous *free1* T-DNA insertional mutant root tips upon BTH treatment for 8hrs using antibodies against ESCRT-III protein SNF7. Arrows indicate examples of the gold-particle labeling on the

autophagosomal structures in wild-type Col-0. Scale bars, 500 nm. **b** Quantification analysis of the gold-particle number on the autophagosomal structures shown in **a**. **c** Confocal analysis of the spatial relationship between SNF7.1-GFP and mCherry-ATG8e in *Arabidopsis* protoplasts expressing FREE1 RNAi, FREE1 RNAi and Myc-FREE1, FREE1 RNAi and Myc-FREE1^{W438AI441A}, or FREE1 RNAi and Myc-FREE1^{S530A}. Arrows and arrowheads indicated the colocalized and separated puncta, respectively. Scale bars, 10 μm. **d** Quantification analysis of the colocalization ratio between SNF7.1 and ATG8e shown in **c**. Means ± SD; n=9 protoplasts per experimental group, one-way analysis of variance (ANOVA) followed by Tukey's multiple test; ***$p<0.001$. **e** Immunoblot analysis of the overexpression of the Myc-FREE1, Myc-FREE1^{W438AI441A}, and Myc-FREE1^{S530A} in *Arabidopsis* protoplasts expressing FREE1 RNAi in **c** using antibodies as indicated. All the imaging analysis and immunoblots were repeated at least for three times with similar results.

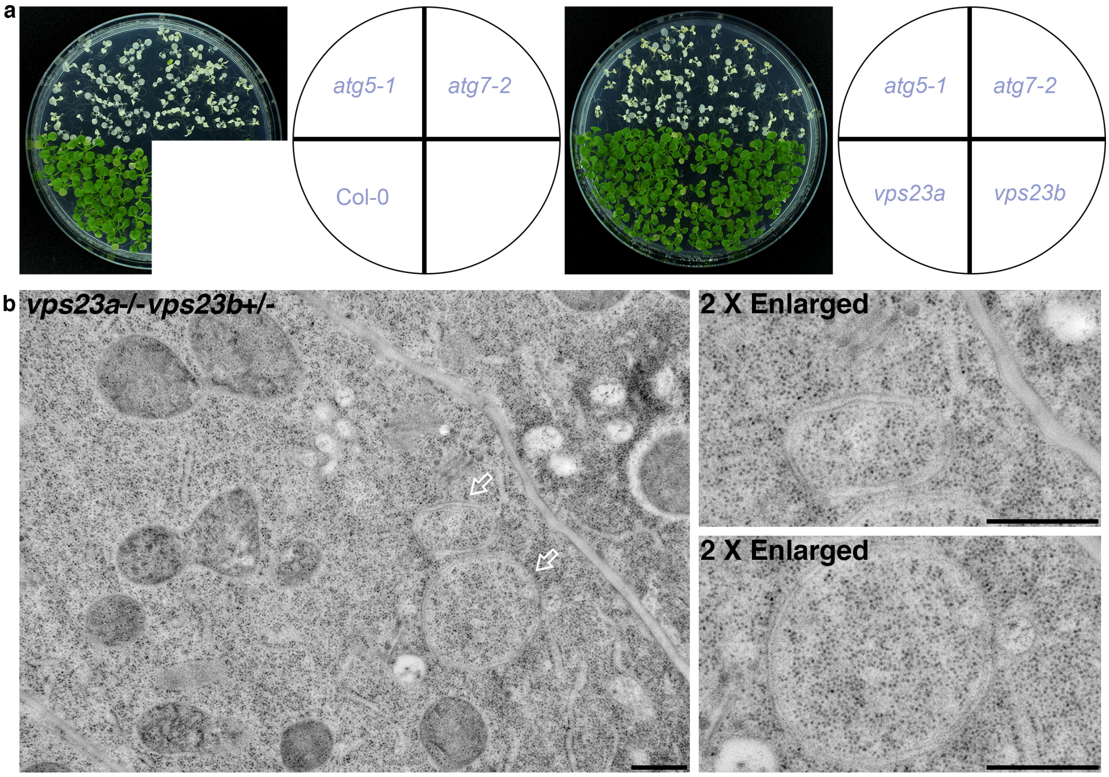

Supplementary Fig 20. *vps23a* or *vps23b* mutants exhibit no obvious defects on plant growth and autophagosome closure upon carbon deprivation. a Phenotypic analysis of Col-0, *atg5-1*, *atg7-2*, *vps23a*, and *vps23b* upon carbon deprivation. **b** Structural TEM analysis of ultrathin sections prepared from high-pressure freezing/frozen substituted *vps23a*-/-*vps23b*+/- plant root tips upon carbon starvation for 18hrs. Arrows indicate examples of the closed autophagosomal structures. Scale bars, 500 nm.

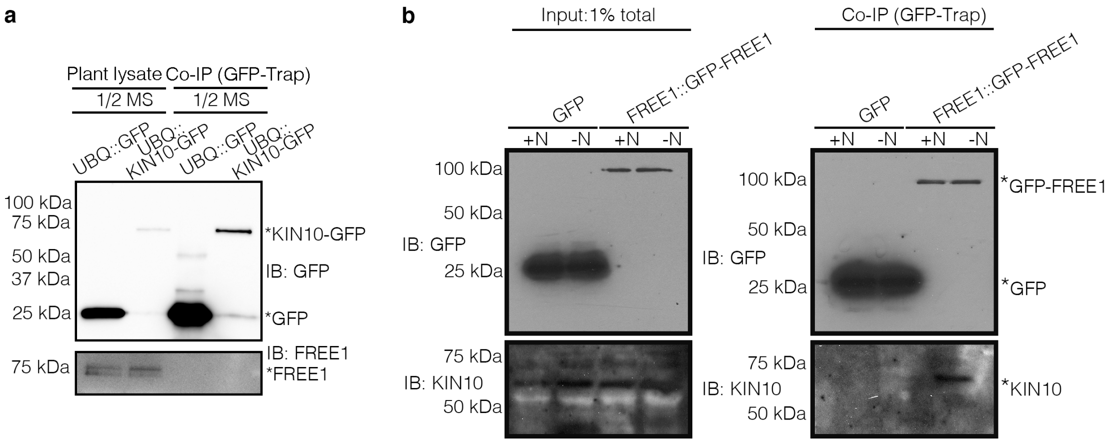

Supplementary Fig 21. FREE1 interacts with endogenous KIN10 *in vivo* upon nitrogen starvation but not under normal growth condition. a GFP-Trap and co-IP analysis of KIN10 and FREE1 using transgenic plants expressing KIN10-GFP under mock condition, followed by immunoblotting analysis using GFP and FREE1 antibodies. **b** GFP-Trap and co-IP analysis of KIN10 and FREE1 using the endogenous promoter driven GFP-FREE1 transgenic plants under normal (+N) or nitrogen starvation (-N) conditions for at least 18hrs, followed by immunoblotting analysis using GFP and KIN10 antibodies. All the immunoblots were repeated at least for three times with similar results.

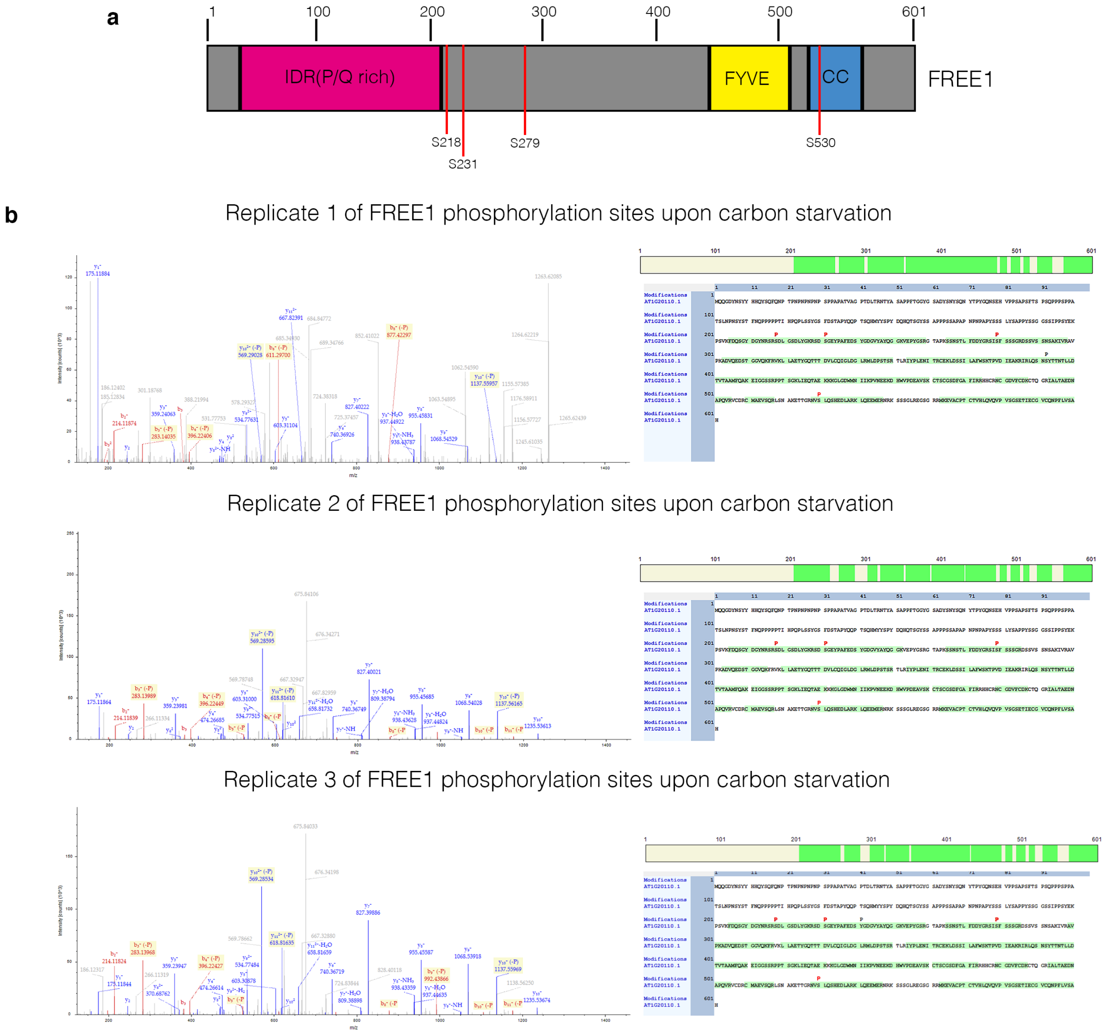

Supplementary Fig 22. Identification of the phosphorylation sites on FREE1 upon carbon starvation. a Schematic diagram of FREE1, showing the potential phosphorylation sites identified from the mass-spectrometry analysis upon carbon starvation. **b** Transgenic *Arabidopsis* seedlings expressing GFP-FREE1 were subjected to carbon starvation for at least 18hrs, followed by protein extraction and IP with GFP-Trap, and subsequent mass-spectrum analysis for phosphorylation sites identification. Raw MS/MS data were converted to Mascot generic format (mgf) and were used to search database using an in-house Mascot search engine (Matrix Science) with phosphorylation. Three replicates were performed and the repeatedly identified phosphorylation sites were highlighted in red.

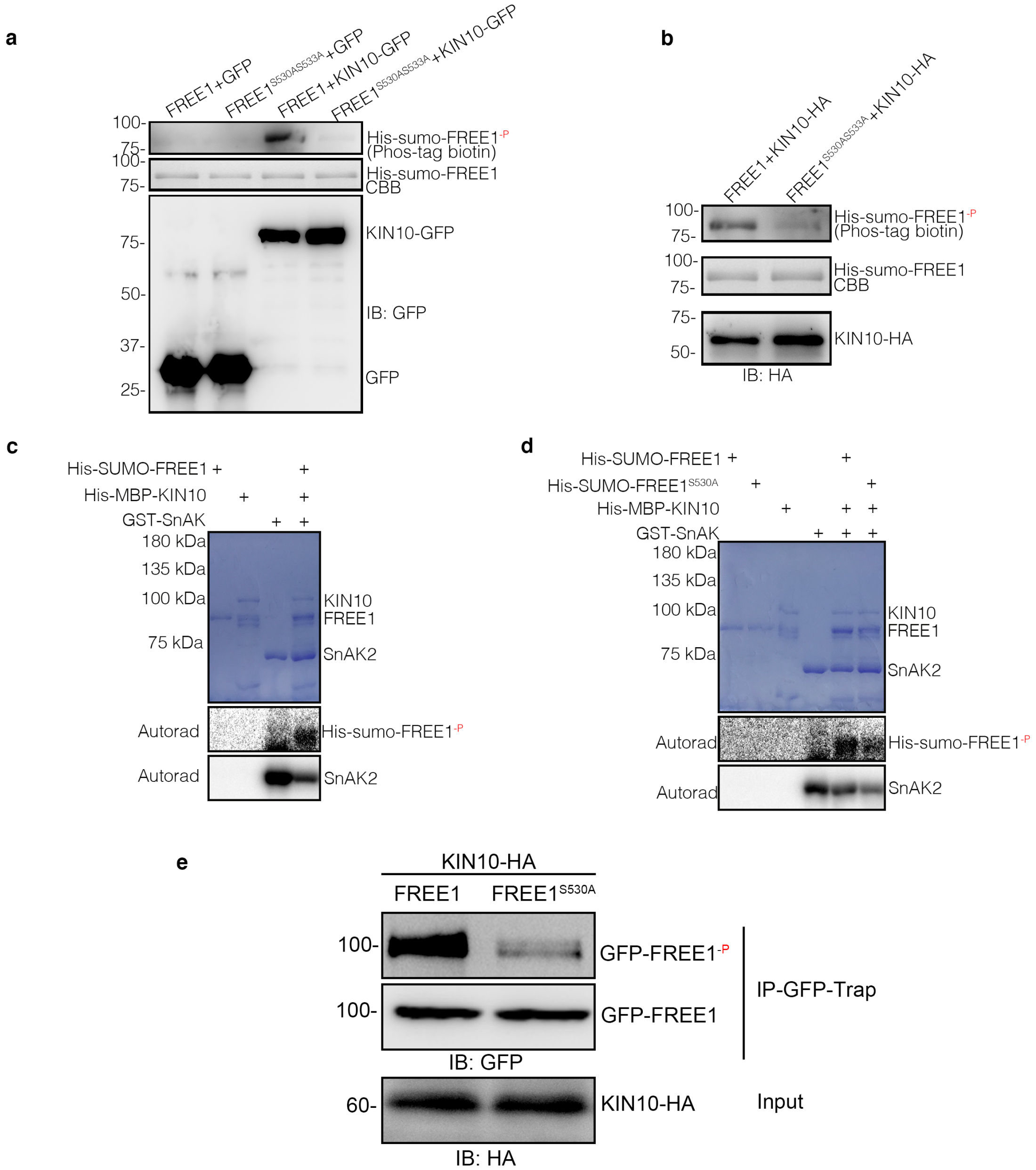

Supplementary Fig 23. *In vitro* and semi-*in vitro* phosphorylation of FREE1 by KIN10. a Semi-*in vitro* phosphorylation assay of FREE1 and FREE1^{S530AS533A} proteins by KIN10-GFP or GFP alone purified from the transgenic plants expressing KIN10-GFP or GFP. KIN10 was enriched from transgenic seedlings expressing KIN10-GFP by immunoprecipitation with anti-GFP and used to phosphorylate FREE1 or FREE1^{S530AS533A} tagged with His-sumo, which was further detected by Phos-tag biotin. **b** Semi-*in vitro* phosphorylation assay of FREE1 and FREE1^{S530AS533A} proteins by KIN10-HA purified from the transgenic plants expressing KIN10-HA. KIN10 was enriched from transgenic seedlings expressing KIN10-HA by immunoprecipitation with anti-HA and used to phosphorylate FREE1 or FREE1^{S530AS533A} tagged with His-sumo, which was further detected by Phos-tag

biotin. **c** *In vitro* phosphorylation assay of FREE1 proteins by KIN10. Purified recombinant KIN10 tagged with His-MBP was incubated with FREE1 tagged with His-sumo in the presence of SnAK2, which was further detected by the autoradiography. **d** *In vitro* phosphorylation assay of FREE1 and FREE1^{S530A} proteins by KIN10. Purified recombinant KIN10 tagged with His-MBP was incubated with FREE1 or FREE1^{S530A} tagged with His-sumo in the presence of SnAK2, which was further detected by the autoradiography. **e** *In planta* phosphorylation of FREE1 proteins by KIN10. GFP-FREE1 or GFP-FREE1^{S530A} were transiently expressed in protoplasts prepared from transgenic plants expressing KIN10-HA upon nitrogen starvation for at least 18hrs. GFP-FREE1 and GFP-FREE1^{S530A} were enriched by immunoprecipitation with anti-GFP for further detection by Phos-tag biotin. All the immunoblots were repeated at least for three times with similar results.

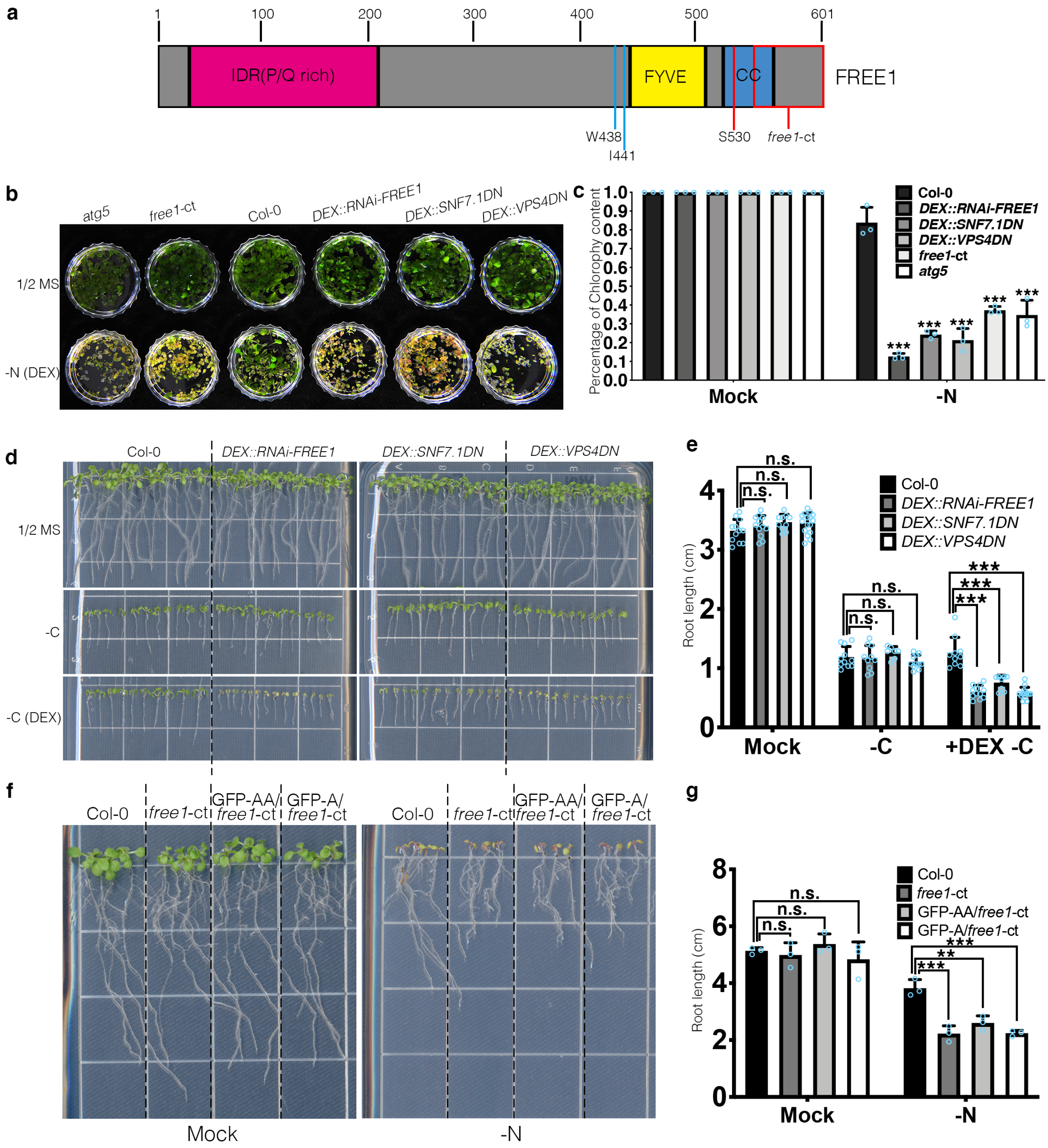

Supplementary Fig 24. Mutants of FREE1 and other ESCRT machinery display autophagy-related phenotypes. **a** Schematic diagram of FREE1, showing the AIM motif, the phosphorylation sites identified from the mass-spectrometry analysis, and the deletion in C-terminus of FREE1 in the CRISPR mutants. **b** Phenotypic analysis of Col-0, *DEX::RNAi-FREE1*, *free1*-ct, *DEX::SNF7.1DN*, *DEX::VPS4DN* and *atg5* mutants upon nitrogen starvation. **c** Chlorophyll content measurement of mutants shown in **b.** Means ± SD; n=3 independent experiments, ≥25 seedlings per genotype were used for analysis in each experiment, two-way analysis of variance (ANOVA) followed by Šídák's multiple comparisons test; ***p<0.001. **d** Phenotypic analysis of Col-0, *DEX::RNAi-FREE1*, *DEX::SNF7.1DN*, and *DEX::VPS4DN* mutants upon carbon deprivation. **e** Quantification of root length of individual genotypes in **c**. Means ± SD; n=12 (mock), n=11 (-C), n=10 (+DEX -C) roots for Col-0, n=12

(mock), n=10 (-C), n=12 (+DEX -C) roots for *DEX::RNAi-FREE1*, n=10 (mock), n=7 (-C), n=8 (+DEX -C) roots for *DEX::SNF7.1DN*, and n=16 (mock), n=11 (-C), n=14 (+DEX -C) roots for *DEX::VPS4DN*, two-way analysis of variance (ANOVA) followed by Tukey's multiple test; ***p<0.001; n.s., not significant. **f** Phenotypic analysis of Col-0, *free1*-ct, GFP-FREE1^{S530AS533A}/*free1*-ct (GFP-AA/*free1*-ct), and GFP-FREE1^{S530A}/*free1*-ct (GFP-A/*free1*-ct) lines upon nitrogen starvation. **g** Quantification of root length of individual genotypes in **f.** Means ± SD; n=3 roots per genotype, two-way analysis of variance (ANOVA) followed by Tukey's multiple test; **p<0.01; ***p<0.001; n.s., not significant.

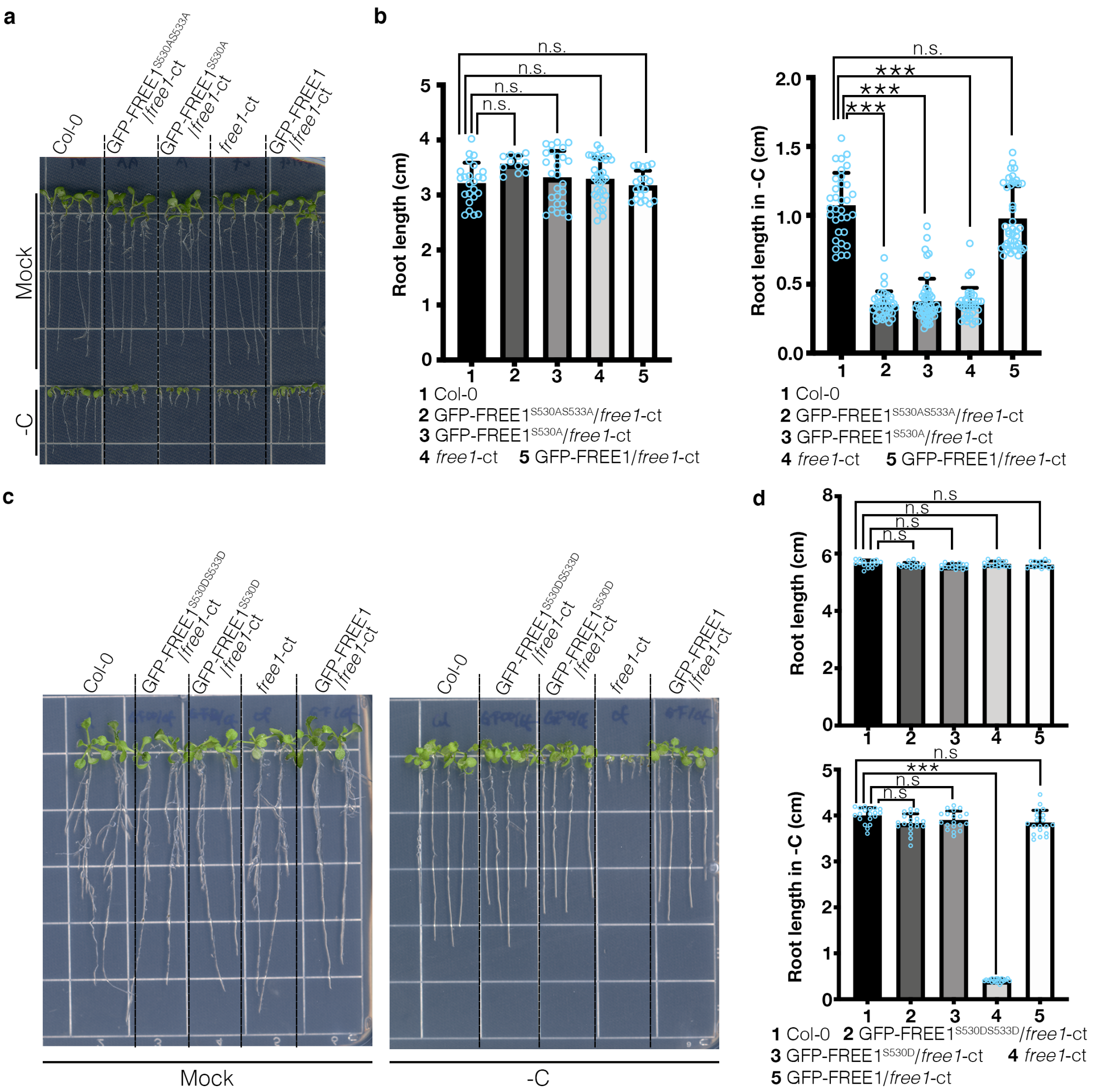

Supplementary Fig 25. S530 is essential for the FREE1 function in plant growth under carbon deprivation condition. a Phenotypic analysis of Col-0, GFP-FREE1^{S530AS533A}/*free1*-ct, GFP-FREE1^{S530A}/*free1*-ct, *free1*-ct, and GFP-FREE1/*free1*-ct upon carbon deprivation and mock condition. **b** Quantification of root length of individual genotypes shown in **a**. Means ± SD; n=24 (mock) and n=33 (-C) roots for Col-0, n=11 (mock) and n=35 (-C) roots for GFP-FREE1^{S530AS533A}/*free1*-ct, n=26 (mock) and n=48 (-C) roots for GFP-FREE1^{S530A}/*free1*-ct, n=32 (mock) and n=35 (-C) roots for *free1*-ct, and n=19 (mock) and n=42 (-C) roots for GFP-FREE1/*free1*-ct, one-way analysis of variance (ANOVA), followed by Tukey's multiple test; ***$p<0.001$; n.s., not significant. **c** Phenotypic analysis of Col-0, GFP-FREE1^{S530DS533D}/*free1*-ct, GFP-FREE1^{S530D}/*free1*-ct, *free1*-ct, and GFP-FREE1/*free1*-ct upon carbon deprivation and mock conditions. **d** Quantification of root length of individual genotypes shown in **c**. Means ± SD; n=15 (mock) and n=19 (-C) roots per

genotypes, one-way analysis of variance (ANOVA), followed by Tukey’s multiple test; ***$p<0.001$; n.s., not significant.

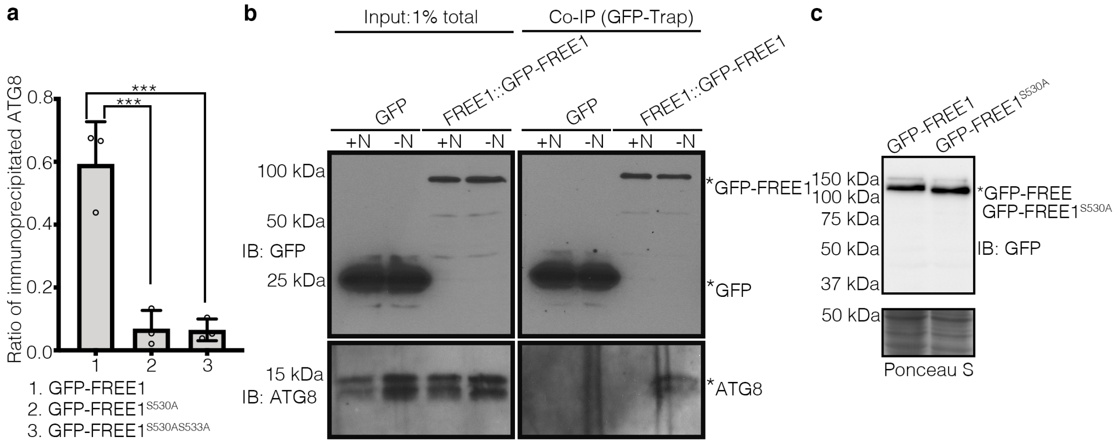

Supplementary Fig 26. FREE1 interacts with endogenous ATG8 under nitrogen starvation condition while S530 mutants abolished the interaction between FREE1 and ATG8. a Quantification analysis of the ratio of immunoprecipitated ATG8 by GFP-FREE1, GFP-FREE1^{S530A}, and GFP-FREE1^{S530AS533A} shown in **Figure 4j**. Means ± SD; n=3 individual experiments, one-way analysis of variance (ANOVA), followed by Tukey's multiple test; ***p<0.001. **b** GFP-Trap and co-IP analysis of ATG8 and FREE1 interaction using the endogenous promoter driven GFP-FREE1 transgenic plants under normal (+N) or nitrogen starvation (-N) conditions for at least 18hrs, followed by immunoblotting detection using the GFP and endogenous ATG8 antibodies. **c** GFP-FREE1/GFP-FREE1^{S530A} is well expressed in *Arabidopsis* protoplasts shown in **Figure 4k**. *Arabidopsis* protoplasts expressing GFP-FREE1 or GFP-FREE1^{S530A} were subjected to protein extraction, followed by immunoblotting with GFP antibody. All the immunoblots were repeated at least for three times with similar results.

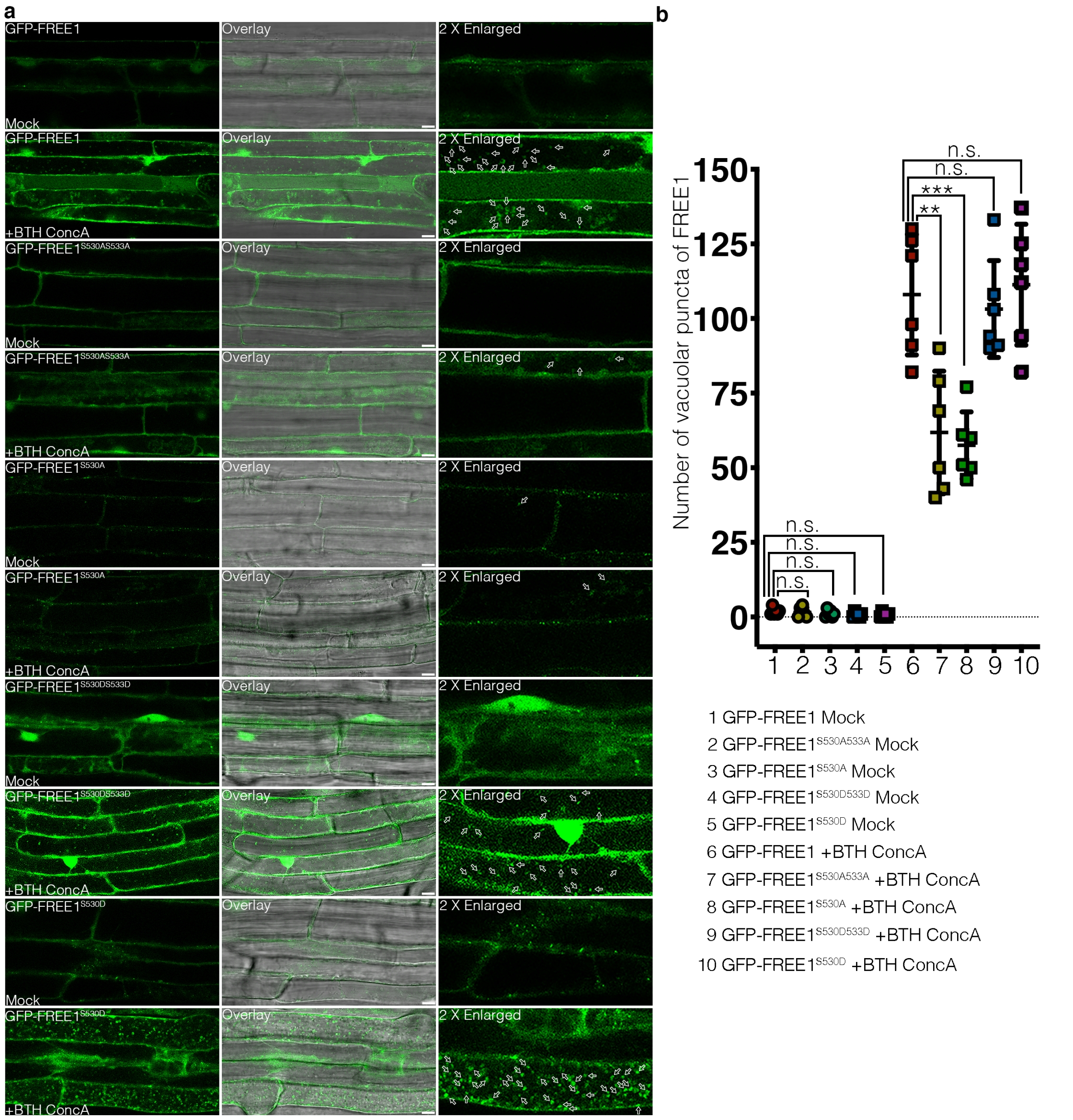

Supplementary Fig 27. S530 is essential for the FREE1 vacuolar turnover. a CLSM analysis of vacuolar delivery of GFP-FREE1, GFP-FREE1^{S530AS533A}, GFP-FREE1^{S530A}, GFP-FREE1^{S530DS533D}, and GFP-FREE1^{S530D} mutant variants upon mock or BTH and ConcA treatments for 8hrs. 2 X Enlarged images showed the vacuolar delivery of GFP-FREE1, GFP-FREE1^{S530AS533A}, and GFP-FREE1^{S530A}, GFP-FREE1^{S530DS533D}, and GFP-FREE1^{S530D} mutant variants, in which arrows indicated the GFP-positive puncta delivered to vacuole. Scale bars, 10 μm. **b** Quantification analysis of the vacuolar delivery of FREE1 and its mutant variants shown in **a**. Means ± SD; n=6 cells per group, one-way analysis of variance (ANOVA), followed by Tukey's multiple test; **p<0.01; ***p<0.001; n.s., not significant. All the imaging analysis was repeated at least for three times with similar results.

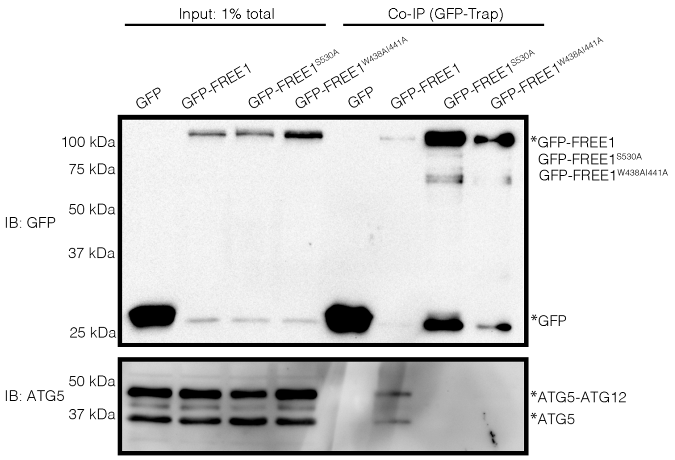

Supplementary Fig 28. S530 on FREE1 is essential for its interaction with endogenous ATG5. GFP-Trap and co-IP of ATG conjugation system components and FREE1 and FREE1 mutant variants using *Arabidopsis* protoplasts, followed by immunoblot analysis using the GFP and endogenous ATG5 antibodies. All the immunoblots were repeated at least for three times with similar results.

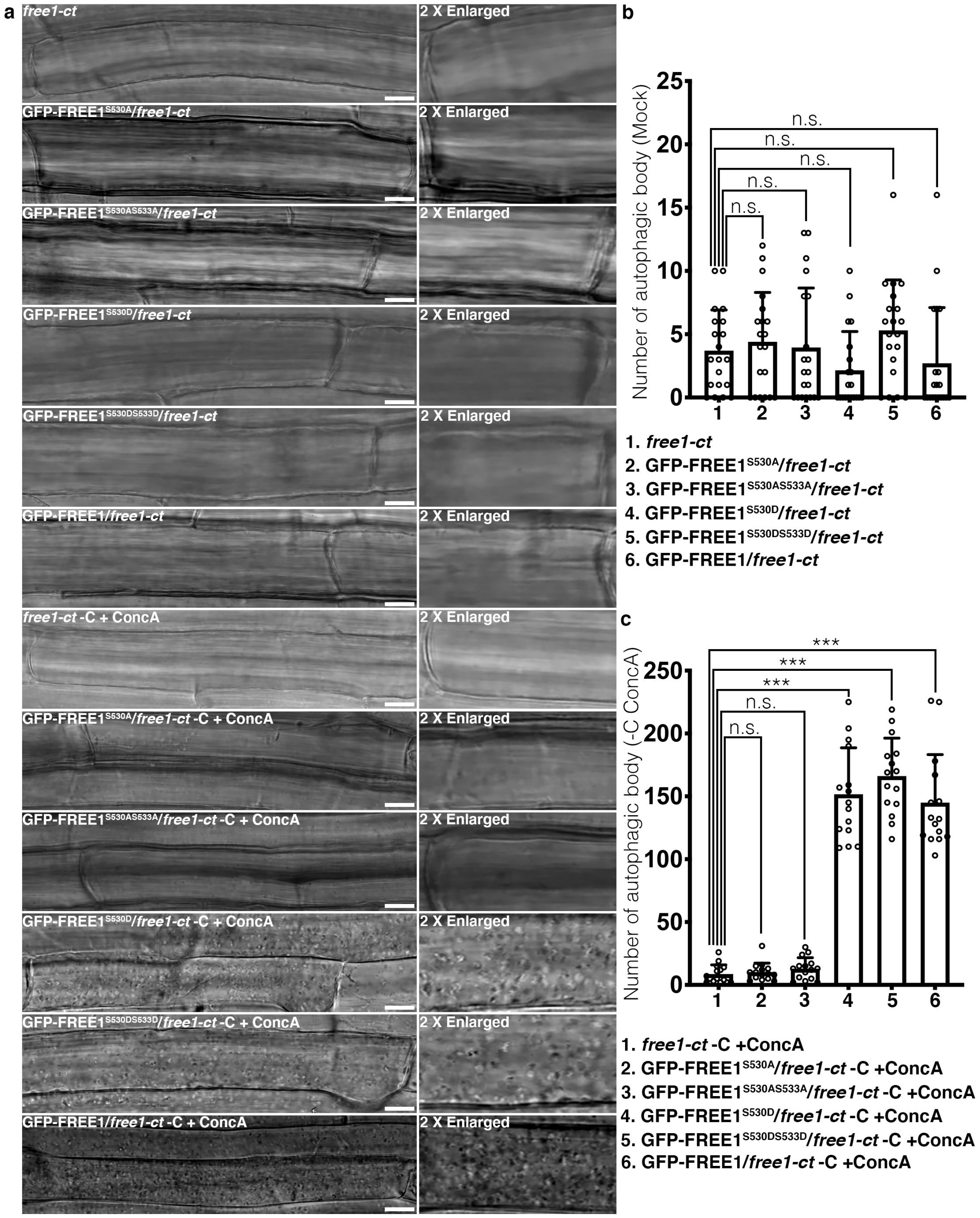

Supplementary Fig 29. The phosphorylation site on FREE1 is essential for the autophagic flux. **a** *free1-ct* mutant, GFP-FREE1^{S530A}/*free1-ct*, GFP-FREE1^{S530AS533A}/*free1-ct*, GFP-FREE1^{S530D}/*free1-ct*, GFP-FREE1^{S530DS533D}/*free1-ct*, and GFP-FREE1/*free1-ct* transgenic plants were subjected to mock or carbon starvation and concanamycin A treatment (-C+ConcA) for at least 18hrs before microscope observation. 2 X Enlarged images indicated the autophagic bodies inside the vacuole in *free1-ct*, GFP-FREE1^{S530A}/*free1-ct*, GFP-FREE1^{S530AS533A}/*free1-ct*, GFP-FREE1^{S530D}/*free1-ct*, GFP-FREE1^{S530DS533D}/*free1-ct*, and GFP-FREE1/*free1-ct* plants. Scale bars, 10 μm. **b-c** Quantifications of the number of autophagic bodies in

vacuoles in **a**. Means ± SD; n=20 (Mock) and n=15 (-C+ConcA) cells per experimental group, one-way analysis of variance (ANOVA), followed by Tukey's multiple test; ***$p<0.001$; n.s., not significant. All the imaging analysis was repeated at least for three times with similar results.

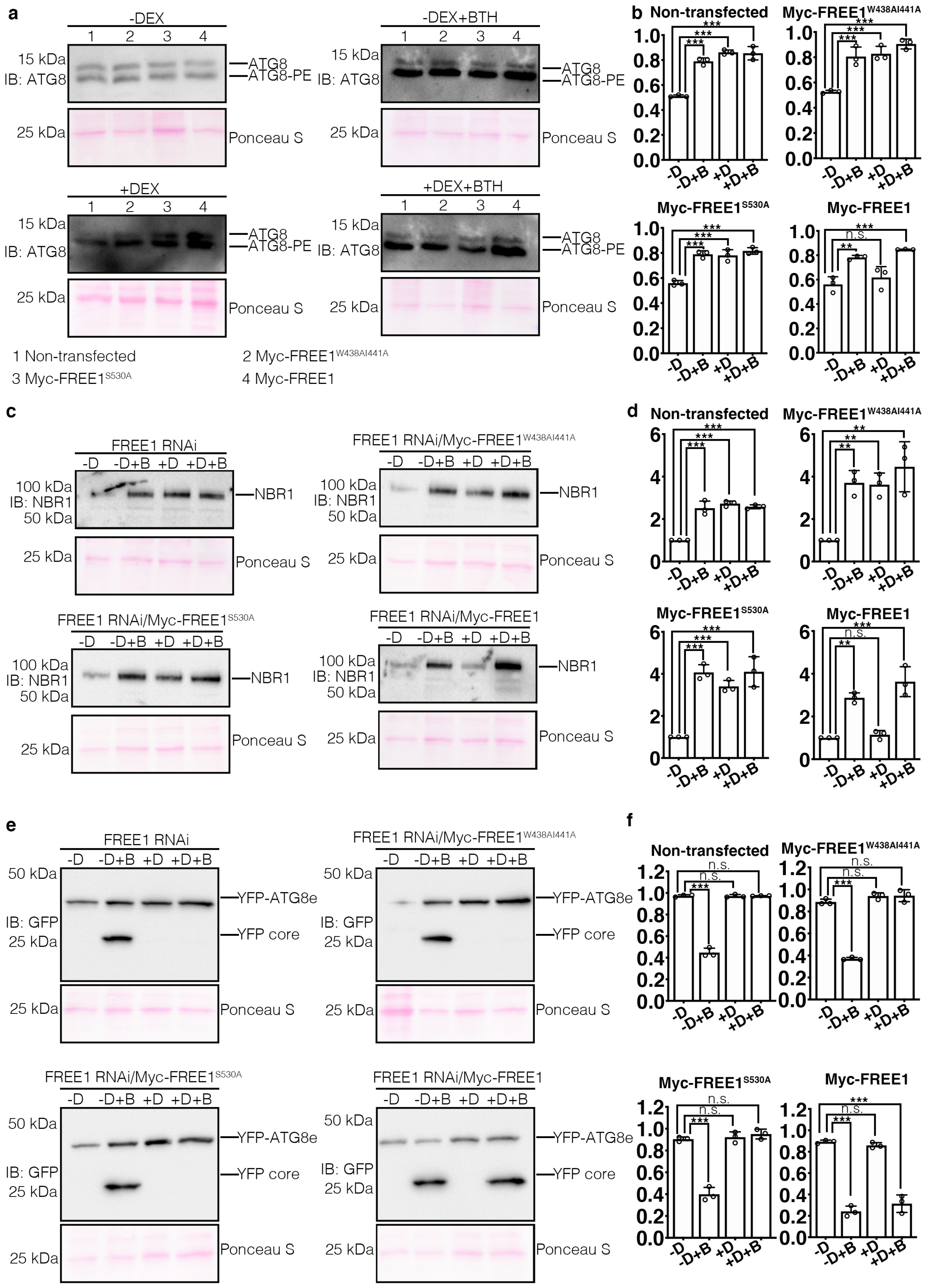

Supplementary Fig 30. LIR motif and S530 are essential for the FREE1 function in autophagy. a Protoplasts isolated from FREE1 RNAi (*DEX::RNAi-FREE1*) mutants were transfected with Myc-FREE1^{W438AI441A} (FREE1 LIRmut), Myc-FREE1^{S530A} or wild-type Myc-FREE1 and treated without DEX (-D), without DEX

with BTH (-D+B), with DEX (+D), or with DEX and BTH (+D+B) for 8hrs, followed by protein extraction and subsequent detection of the ATG8 lipidation using Atg8 antibody. **b** Quantification analysis of the ATG8-PE ratio in individual experimental group. Means ± SD; n=3 individual experiments, one-way analysis of variance (ANOVA), followed by Tukey's multiple test; **p<0.01; ***p<0.001; n.s., not significant. **c** Protoplasts isolated from FREE1 RNAi (*DEX::RNAi-FREE1*) mutants were transfected with Myc-FREE1^{W438AI441A} (FREE1 LIRmut), Myc-FREE1^{S530A} or wild-type Myc-FREE1 and treated without DEX (-D), without DEX with BTH (-D+B), with DEX (+D), or with DEX and BTH (+D+B) for 8hrs, followed by protein extraction and immunoblot detection of the endogenous NBR1 accumulation by NBR1 antibody. **d** Quantification analysis of the NBR1 ratio in each group comparing with the control (-D) in individual experimental group. Means ± SD; n=3 individual experiments, one-way analysis of variance (ANOVA), followed by Tukey's multiple test; **p<0.01; ***p<0.001; n.s., not significant. **e** Protoplasts isolated from transgenic *Arabidopsis* seedlings expressing YFP-ATG8e in FREE1 RNAi (*DEX::RNAi-FREE1*) mutants were transfected with Myc-FREE1^{W438AI441A} (FREE1 LIRmut), Myc-FREE1^{S530A} or wild-type Myc-FREE1 and treated without DEX (-D), without DEX with BTH (-D+B), with DEX (+D), or with DEX and BTH (+D+B) for 8hrs, followed by protein extraction and immunoblot detection by GFP antibody. **f** Quantification analysis of the full-length YFP-ATG8e ratio in individual experimental group. Means ± SD; n=3 individual experiments, one-way analysis of variance (ANOVA), followed by Tukey's multiple test; ***p<0.001; n.s., not significant. All the immunoblots were repeated at least for three times with similar results.

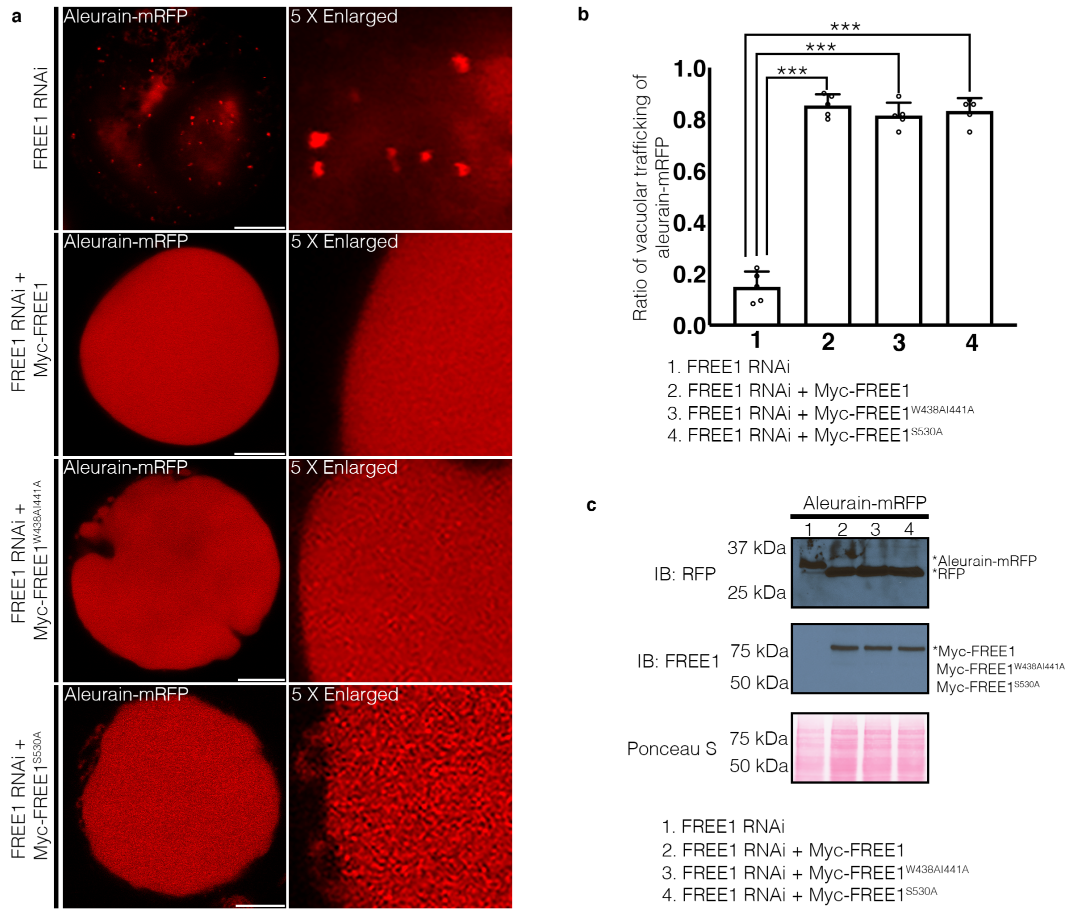

Supplementary Fig 31. LIR motif and S530 do not affect the vacuolar function of FREE1. a Confocal analysis of the vacuolar transport of the vacuolar cargo Aleurain-mRFP in *Arabidopsis* protoplasts expressing FREE1 RNAi, FREE1 RNAi and Myc-FREE1, FREE1 RNAi and Myc-FREE1^{W438AI441A}, or FREE1 RNAi and Myc-FREE1^{S530A}. Scale bars, 10 μm. **b** Quantification analysis of the ratio of protoplasts showing vacuole pattern of Aleurain-mRFP shown in **a**. Means ± SD; n=5 individual experiments, one-way analysis of variance (ANOVA) followed by Tukey's multiple test; ***p<0.001. **c** Immunoblot analysis of the vacuolar transport of the vacuolar cargo Aleurain-mRFP in *Arabidopsis* protoplasts expressing FREE1 RNAi, FREE1 RNAi and Myc-FREE1, FREE1 RNAi and Myc-FREE1^{W438AI441A}, or FREE1 RNAi and Myc-FREE1^{S530A} using antibodies as indicated. All the imaging analysis and immunoblots were repeated at least for three times with similar results.
