# Supplementary material for: The plant unique ESCRT component FREE1 regulates autophagosome closure
Source: Nat Commun. 2023 Mar 30;14:1768. doi: 10.1038/s41467-023-37185-6 (PMC10063618; doi:10.1038/s41467-023-37185-6)
Supplement: Supplementary file 3 — Description of Additional Supplementary Files [file 41467_2023_37185_MOESM3_ESM.pdf]

Description of Additional Supplementary Files:

Supplementary Movie 1. Live-cell imaging of APs formation in Col-0 upon autophagic induction. (Scale bar, 10 μm)

Supplementary Movie 2. Live-cell imaging of unsealed APs in DEX::RNAi-FREE1 upon autophagic induction. (Scale bar, 10 μm)

Supplementary Movie 3. 3D tomography observation of unsealed APs in free1 mutant upon autophagic induction.

Supplementary Movie 4. 3D tomography observation of unsealed APs in atg5-1 mutant upon autophagic induction.

Supplementary Movie 5. 3D tomography observation of unsealed APs in DEX::SNF7.1DN upon autophagic induction.

Supplementary Movie 6. 3D tomography observation of unsealed APs in FREE1 phosphorylation site mutants upon autophagic induction.

Supplementary Data 1. FREE1 interactome analysis upon nutrient starvation.

Supplementary Data 2. All primers and constructs used in this study.
