# Supplementary material for: The plant unique ESCRT component FREE1 regulates autophagosome closure
Source: Nat Commun. 2023 Mar 30;14:1768. doi: 10.1038/s41467-023-37185-6 (PMC10063618; doi:10.1038/s41467-023-37185-6)
Supplement: Supplementary file 12 — Reporting Summary [file 41467_2023_37185_MOESM12_ESM.pdf]

nature portfolio

Corresponding author(s): Liwen JIANG

Last updated by author(s): Feb 14, 2023

Reporting Summary

Nature Portfolio wishes to improve the reproducibility of the work that we publish. This form provides structure for consistency and transparency in reporting. For further information on Nature Portfolio policies, see our Editorial Policies and the Editorial Policy Checklist.

Statistics

For all statistical analyses, confirm that the following items are present in the figure legend, table legend, main text, or Methods section.

n/a	Confirmed	
☐	☒	The exact sample size (*n*) for each experimental group/condition, given as a discrete number and unit of measurement
☐	☒	A statement on whether measurements were taken from distinct samples or whether the same sample was measured repeatedly
☐	☒	The statistical test(s) used AND whether they are one- or two-sided<br>*Only common tests should be described solely by name; describe more complex techniques in the Methods section.*
☒	☐	A description of all covariates tested
☒	☐	A description of any assumptions or corrections, such as tests of normality and adjustment for multiple comparisons
☐	☒	A full description of the statistical parameters including central tendency (e.g. means) or other basic estimates (e.g. regression coefficient) AND variation (e.g. standard deviation) or associated estimates of uncertainty (e.g. confidence intervals)
☐	☒	For null hypothesis testing, the test statistic (e.g. *F*, *t*, *r*) with confidence intervals, effect sizes, degrees of freedom and *P* value noted<br>*Give P values as exact values whenever suitable.*
☒	☐	For Bayesian analysis, information on the choice of priors and Markov chain Monte Carlo settings
☒	☐	For hierarchical and complex designs, identification of the appropriate level for tests and full reporting of outcomes
☒	☐	Estimates of effect sizes (e.g. Cohen's *d*, Pearson's *r*), indicating how they were calculated

Our web collection on statistics for biologists contains articles on many of the points above.

Software and code

Policy information about availability of computer code

Data collection	Leica SP8 laser scanning confocal system, Nikon NSIM system, Hitachi H-7650 transmission electron microscope, 200 kV FEI Tecnai F20 transmission electron microscope, Thermo Fisher Scientific Orbitrap Fusion Lumus mass spectrometer
Data analysis	Image J, HyVolution (Leica), GraphPad Prism version 7.00 for mac (GraphPad Software Inc.), etomo and 3dmod program of the IMOD software package

For manuscripts utilizing custom algorithms or software that are central to the research but not yet described in published literature, software must be made available to editors and reviewers. We strongly encourage code deposition in a community repository (e.g. GitHub). See the Nature Portfolio guidelines for submitting code & software for further information.

Data

Policy information about availability of data

All manuscripts must include a data availability statement. This statement should provide the following information, where applicable:

- Accession codes, unique identifiers, or web links for publicly available datasets
- A description of any restrictions on data availability
- For clinical datasets or third party data, please ensure that the statement adheres to our policy

The authors declare that the data supporting the findings of this study are available in the paper and its supplementary information files. The raw data are available from the corresponding author upon reasonable request. Source data are provided with this paper.

Human research participants

Policy information about studies involving human research participants and Sex and Gender in Research.

Reporting on sex and gender	*Use the terms sex (biological attribute) and gender (shaped by social and cultural circumstances) carefully in order to avoid confusing both terms. Indicate if findings apply to only one sex or gender; describe whether sex and gender were considered in study design whether sex and/or gender was determined based on self-reporting or assigned and methods used. Provide in the source data disaggregated sex and gender data where this information has been collected, and consent has been obtained for sharing of individual-level data; provide overall numbers in this Reporting Summary. Please state if this information has not been collected. Report sex- and gender-based analyses where performed, justify reasons for lack of sex- and gender-based analysis.*
Population characteristics	*Describe the covariate-relevant population characteristics of the human research participants (e.g. age, genotypic information, past and current diagnosis and treatment categories). If you filled out the behavioural & social sciences study design questions and have nothing to add here, write "See above."*
Recruitment	*Describe how participants were recruited. Outline any potential self-selection bias or other biases that may be present and how these are likely to impact results.*
Ethics oversight	*Identify the organization(s) that approved the study protocol.*

Note that full information on the approval of the study protocol must also be provided in the manuscript.

Field-specific reporting

Please select the one below that is the best fit for your research. If you are not sure, read the appropriate sections before making your selection.

☒ Life sciences ☐ Behavioural & social sciences ☐ Ecological, evolutionary & environmental sciences

For a reference copy of the document with all sections, see nature.com/documents/nr-reporting-summary-flat.pdf

Life sciences study design

All studies must disclose on these points even when the disclosure is negative.

Sample size	No statistical method was used to predetermine the sample sizes. Sample sizes were chosen as large as possible while still practically doable in terms of data collection and as sufficient when results could be reliably reproduced.<br><br>For Co-IP and Western blotting analyses, at least three biological replicates were performed. Each replicate contained a group of Arabidopsis seedlings weighing around 0.5 g to 2 g.<br><br>For confocal observations, at least three biological replicates were performed. More than 5 individual seedlings, 10 individual root cells, or 10 protoplast cells were observed for each biological replicate.<br><br>For phenotypic analysis upon carbon deprivation, three biological replicates were performed. More than 30 seedlings of each genotype were used for analysis in each biological replicate.<br><br>For phenotypic analysis and chlorophyll content measurement upon nitrogen starvation, three biological replicates were performed. More than 25 seedlings of each genotype were used for analysis in each biological replicate.<br><br>For root length analysis upon carbon deprivation, three biological replicates were performed. More than 7 seedlings of each genotype were used for analysis in each biological replicate.<br><br>For root length analysis upon nitrogen starvation, three biological replicates were performed. More than 3 seedings of each genotype were used for analysis in each biological replicate.<br><br>For TEM and 3D EM tomography study, more than 15 Arabidopsis root tips of each genotpye were randomly selected for high pressure freezing. A minimum of three blocks (each block contains 1-2 root tips) from each sample were randomly selected for sectioning and TEM imaging. More than 5 root tips or 10 root cells of each sample were randomly selected for statistical analysis in each biological replicates.<br><br>For IEM analysis, more than 10 cells per group were randomly selected for statistical analysis in each biological replicates.
Data exclusions	No data were excluded from the analysis.
Replication	The experimental findings were reliably reproduced at least three times in this study.

Randomization	The Arabidopsis seedlings of different genotypes were randomly selected and used for different treatments. The Arabidopsis seedlings or protoplasts were randomly selected for confocal observation. The high-pressure frozen roots were randomly selected for TEM observation and IEM analysis.
Blinding	The investigators were blinded to group allocation during data collection and analysis.

Reporting for specific materials, systems and methods

We require information from authors about some types of materials, experimental systems and methods used in many studies. Here, indicate whether each material, system or method listed is relevant to your study. If you are not sure if a list item applies to your research, read the appropriate section before selecting a response.

Materials & experimental systems

n/a	Involved in the study	
☐	☒	Antibodies
☒	☐	Eukaryotic cell lines
☒	☐	Palaeontology and archaeology
☒	☐	Animals and other organisms
☒	☐	Clinical data
☒	☐	Dual use research of concern

Methods

n/a	Involved in the study	
☒	☐	ChIP-seq
☒	☐	Flow cytometry
☒	☐	MRI-based neuroimaging

Antibodies

Antibodies used

GFP antibody (Rabbit), FREE1 antibody, and NBR1 antibody were home-made as described in our previous publication. SNF7 antibody was a gift from Prof. Marisa S Otegui from University of Wisconsin-Madison. Additional primary antibodies used were commercially available (anti-Myc (Cat no SC-789) from Santa Cruz, anti-HA (Cat no ab18181) from Abcam, anti-GFP (Cat no 3H9) and anti-RFP (Cat no 5F8) from Chromotek, anti-KIN10 (Product no AS10 919), anti-ATG8 (Product no AS14 2769), anti-ATG1a (Product no AS19 4274), anti-ATG13a (Product no AS19 4279), anti-ATG5 (AS15 3060), anti-ATG12b (AS19 4278), and anti-ATG16 (AS19 4280) from Agrisera).

Validation

Home-made GFP antibody (Rabbit) was described and validated in previous publication (Cui et al., Nature Plants, 2019, 5, 95–105).

Home-made FREE1 antibody was described and validated in previous publication (Gao et al., Current Biology, 2014, 24(21), 2556-2563).

Home-made NBR1 antibody was described and validated in previous publication (Ji et al., Plant Physiology, 2020, 184(2), 777-791).

SNF7 antibody was a gift as described and validated in previous publication (Buono et al., J Cell Biol, 2017, 216(7), 2167-2177).

Validation statement for Myc antibody (Cat no SC-789) can be found at the product website. <https://www.scbt.com/p/c-myc-antibody-a-14?requestFrom=search>

Validation statement for HA antibody (Cat no ab18181) can be found at the product website. <https://www.abcam.com/ha-tag-antibody-hac5-ab18181.html>

Validation statement for GFP antibody (Cat no 3H9) can be found at the product website. <https://www.ptglab.com/products/GFP-antibody-3H9.htm>

Validation statement for RFP antibody (Cat no 5F8) can be found at the product website. <https://www.ptglab.com/products/RFP-antibody-5F8.htm>

Validation statement for KIN10 antibody (Product no AS10 919) can be found at the product website. <https://www.agrisera.com/en/artiklar/akin10-snf1-related-protein-kinase-catalytic-subunit-alpha-kin10.html>

Validation statement for ATG8 antibody (Product no AS14 2769) can be found at the product website. <https://www.agrisera.com/en/artiklar/atg8.html>

Validation statement for ATG1a antibody (Product no AS19 4274) can be found at the product website. <https://www.agrisera.com/en/artiklar/atg1a.html>

Validation statement for ATG13a antibody (Product no AS19 4279) can be found at the product website. <https://www.agrisera.com/en/artiklar/atg13a.html>

Validation statement for ATG5 antibody (Product no AS15 3060) can be found at the product website. <https://www.agrisera.com/en/artiklar/atg5-autophagy-related-protein-5.html>

Validation statement for ATG12b antibody (Product no AS19 4278) can be found at the product website. <https://www.agrisera.com/en/artiklar/atg12b.html>

Validation statement for ATG16 antibody (Product no AS19 4280) can be found at the product website.<https://www.agrisera.com/en/artiklar/atg16.html>
